# Unraveling signatures of chicken genetic diversity and divergent selection in breed-specific patterns of early myogenesis, nitric oxide metabolism and post-hatch growth

**DOI:** 10.3389/fgene.2022.1092242

**Published:** 2023-01-11

**Authors:** Ivan I. Kochish, Vladimir Yu. Titov, Ilya N. Nikonov, Evgeni A. Brazhnik, Nikolai I. Vorobyov, Maxim V. Korenyuga, Olga V. Myasnikova, Anna M. Dolgorukova, Darren K. Griffin, Michael N. Romanov

**Affiliations:** ^1^ K. I. Skryabin Moscow State Academy of Veterinary Medicine and Biotechnology, Moscow, Russia; ^2^ Federal Scientific Center “All-Russian Poultry Research and Technological Institute” of the Russian Academy of Sciences, Sergiev Posad, Moscow Oblast, Russia; ^3^ BIOTROF+ Ltd., Pushkin, St. Petersburg, Russia; ^4^ All-Russia Institute for Agricultural Microbiology, Pushkin, St. Petersburg, Russia; ^5^ School of Biosciences, University of Kent, Canterbury, United Kingdom

**Keywords:** chicken, genetic diversity, divergent selection, breeds, early myogenesis, differential gene expression, nitric oxide oxidation, post-hatch growth

## Abstract

**Introduction:** Due to long-term domestication, breeding and divergent selection, a vast genetic diversity in poultry currently exists, with various breeds being characterized by unique phenotypic and genetic features. Assuming that differences between chicken breeds divergently selected for economically and culturally important traits manifest as early as possible in development and growth stages, we aimed to explore breed-specific patterns and interrelations of embryo myogenesis, nitric oxide (NO) metabolism and post-hatch growth rate (GR).

**Methods:** These characteristics were explored in eight breeds of different utility types (meat-type, dual purpose, egg-type, game, and fancy) by incubating 70 fertile eggs per breed. To screen the differential expression of seven key myogenesis associated genes (*MSTN*, *GHR*, *MEF2C*, *MYOD1*, *MYOG*, *MYH1*, and *MYF5*), quantitative real-time PCR was used.

**Results:** We found that myogenesis associated genes expressed in the breast and thigh muscles in a coordinated manner showing breed specificity as a genetic diversity signature among the breeds studied. Notably, coordinated (“accord”) expression patterns of *MSTN*, *GHR*, and *MEFC2* were observed both in the breast and thigh muscles. Also, associated expression vectors were identified for *MYOG* and *MYOD1* in the breast muscles and for *MYOG* and *MYF5* genes in the thigh muscles. Indices of NO oxidation and post-hatch growth were generally concordant with utility types of breeds, with meat-types breeds demonstrating higher NO oxidation levels and greater GR values as compared to egg-type, dual purpose, game and fancy breeds.

**Discussion:** The results of this study suggest that differences in early myogenesis, NO metabolism and post-hatch growth are breed-specific; they appropriately reflect genetic diversity and accurately capture the evolutionary history of divergently selected chicken breeds.

## 1 Introduction

As a relatively inexpensive source of quality animal protein in the form of meat and eggs, the rearing and use of poultry is a very important livestock production sector (Bogolyubsky, 1991; Romanov et al., 2009). Over several millennia of domestication, breeding and selection, various poultry breeds and varieties have been created by humans that are adapted to certain conditions of keeping and exploited economically for meat, eggs and other purposes (e.g., cock fighting and aesthetic needs). These breeds present a variety of phenotypic traits and, accordingly, can be classified based on their origin, phenotypes, selection targets and utility purpose (Abdelmanova et al., 2021; Larkina et al., 2021; Romanov et al., 2021). According to the traditional classification model (Bogolyubsky, 1991), the main classes of chicken breeds are meat, egg, dual purpose (i.e., meat-egg and egg-meat), game, and fancy (or ornamental). Moiseyeva et al. (2003) postulated an evolutionary model of breed formation with four main branches: egg (Mediterranean), meat (Asian), game, and Bantam ones. Larkina et al. (2021) proposed a phenotypic clustering model, supplementing the evolutionary model with two more breed types, i.e., dual purpose and fancy breeds (see breed examples in Table 1; Supplementary Material S2). Assessment of genetic diversity in various breeds is an important element in developing new strategies and applications for poultry breeding and production, as well as germplasm preservation (Romanov and Weigend, 1999; 2001; Huang et al., 2016; Romanov et al., 2017; Romanov et al., 2021; Bernini et al., 2021; Dementieva et al., 2021).

**TABLE 1:** Characterization of the studied chicken breeds.

Breed	Code	Type of divergent selection			Origin	Description
		TCM^a^	EM^b^	PCM^c^		
Broiler	BR	Meat	Meat	Meat	Russia	4-way BR cross Smena 8 developed in 2011 at the Breeding Genetic Center “Smena”—Branch of the Federal Scientific Center “All-Russian Poultry Research and Technological Institute” of the Russian Academy of Sciences
White Cornish	WC	Meat	Meat	Meat	Russia/England	B56, male grandparent stock, of BR cross Smena 8. The initial breed was developed from English local game chickens, Asil, White Malay, Indian Game, and Cochin
Plymouth Rock White	PRW	Dual purpose (meat-egg type)	Meat	Dual purpose (meat-egg type)	Russia/United States	B79, female grandparent stock, of BR cross Smena 8. The initial breed was developed from Java Black, Brahma, Cochin White and Buff, Dominique, and White-faced Black Spanish
Yurlov Crower	YC	Dual purpose (meat-egg type)	Meat	Dual purpose (meat-egg type)	Russia	Derived in 19th century from crossing local and game chickens, Brahma, Cochin, and Langshan. Selected for long crowing
Brahma Buff	BB	Fancy	Meat	Dual purpose (egg-meat type)	USA/India	Derived in early 20th century from crossing Cochin and Gray Chittagong (of Malay type)
Orloff Mille Fleur	OMF	Fancy/Game	Game	Dual purpose (meat-egg type)	Russia	Derived in late 18th century from crossing local chickens, Gilan, and Old English Game
Layer	LR	Egg	Egg	Egg	Netherlands	Commercial 4-way layer cross Hisex White
Uzbek Game (Kulangi)	UG	Game	Game	Game	Uzbekistan	An old cock fighting breed derived from local Uzbek game chickens

Chicken breed types according to:

^a^ TCM, traditional classification model (Bogolyubsky, 1991).

^b^ EM, evolutionary model (Moiseyeva et al., 2003).

^c^ PCM, phenotypic clustering model (Larkina et al., 2021).

The manifestation of differences between divergently selected and genetically diverse poultry types and breeds can be expected at the earliest stages of embryonic and postnatal development. First of all, this can be traced by the breed-specific features of early myogenesis in embryos and postnatal growth in chicks as was shown by Kanakachari et al. (2022). However, that study included only a broiler line and a native Indian breed. Therefore, it would be reasonable and purposeful to establish the respective genetic diversity signatures by comparing differential gene expression (DGE) among genes responsible for myogenesis in muscle tissues in a broader sample of various chicken breeds divergently selected for meat and egg performance and other phenotypic traits.

Cazzato et al. (2014) studied the earliest stages of embryogenesis and DGE for five key genes controlling the course of skeletal muscle development, such as myosin (*MYH1*; e.g., Thompson et al., 2021) and related ones. As shown by Cazzato et al. (2014), there are effects of nitric oxide synthase inhibitor (NOSI) and nitric oxide donor (NOD) compounds on DGE of myogenesis associated genes. To date, more evidence has been accumulated regarding the role of nitric oxide (NO) in embryogenesis and, in particular, myogenesis (e.g., Cazzato et al., 2014; Titov et al., 2018; Titov et al., 2020b; Titov et al., 2021; Dolgorukova et al., 2020). NO is believed to mediate myocyte proliferation (Ulibarri et al., 1999; Stamler and Meissner, 2001; Long et al., 2006; Li et al., 2016; Tirone et al., 2016), muscle fiber formation (Stamler and Meissner, 2001; Long et al., 2006), and satellite cell proliferation (Anderson, 2000). In conformity with current concepts, the physiological effect of NO manifests itself through the nitrosation of certain protein structures/enzymes: guanylate cyclase (Stamler et al., 1992; Severina et al., 2003), caspases (Dimmeler et al., 1997; Rossig et al., 1999; Kim et al., 2000), as well as molecular cellular factors that determine transcriptional regulation and DGE (Zhou and Brüne, 2005; Vasudevan et al., 2016; Socco et al., 2017).

To evaluate the role of NO in a particular physiological process, a monitoring technique for its synthesis and metabolism to the final product, nitrate, is needed. Synthesized NO is included in NOD compounds (Titov et al., 2020a): S-nitrosothiols (RSNO), dinitrosyl-iron complexes (DNIC), and high molecular weight nitro derivatives (RNO_2_). These compounds play the role of physiological depots of NO, prolonging its physiological lifetime (Severina et al., 2003; Vanin et al., 2017; Vanin et al., 2017). Their concentration in cells can reach tens of µM (Hickok et al., 2011; Titov et al., 2016). Therefore, to determine the NO role in a specific process (e.g., embryogenesis), it is necessary to monitor changes in the content of deposited NO and its metabolic products during this process. It is not straightforward to precisely detect the content of all NO metabolites in living tissues, e.g., methods for determining DNIC and RSNO do not have high accuracy and specificity (Tarpey et al., 2004; Titov, 2011; Vanin et al., 2017; Vanin et al., 2017). To address this problem, conclusions about the effect of NO on a particular physiological process can be inferred based on the effects of NOSI, NOD compounds, and arginine, which is a source of NO (Stamler et al., 1992; Anderson, 2000; Long et al., 2006; Cazzato et al., 2014).

Previously, we developed an enzymatic sensory method that is based on reversible inhibition of catalase by all nitroso compounds and enables detecting the concentration of RSNO, DNIC, nitrite, and nitrosamines with an accuracy of 50 nM (Titov, 2011; Titov et al., 2016). Using this sensor, we confirmed an assumption that DNIC are the main NOD in most tissues (Titov et al., 2016; Titov et al., 2018). It was shown that the embryogenesis of birds, like in other animals, is associated with intense production of NO that either accumulates in the embryo as part of NOD compounds or is oxidized to nitrate. NO oxidation proceeds throughout the entire embryonic period. Within the same species, the intensity of NO synthesis is approximately the same but there are differences in the degree of NO oxidation to nitrate. The latter indicator, according to our previous observations (Titov et al., 2018; Titov et al., 2021), can be many times higher in meat-type chickens than in egg-type breeds. Post hatch, the concentration of nitro compounds and nitroso compounds in the chick tissues declines sharply as compared with the embryo tissues and levels off in various breeds, lines, and crosses (Titov et al., 2018). Analysis of the content of nitro compounds and nitroso compounds in various embryo tissues showed that nitrate mainly accumulates in muscle tissue. It does not accumulate in the liver and intestines to any great degree (Titov et al., 2018), and apparently, NO oxidation mainly occurs in the muscle tissue.

The present study aimed to explore signatures of chicken genetic diversity and divergent selection by examining breed-specific patterns of early myogenesis (assessed by DGE of myogenesis associated genes) and post-hatch growth in various breeds. Therewith, one of the objectives was also to investigate mechanisms of the relationship between the utility type of chicken breeds and intensity of NO oxidation in embryos (i.e., in their muscle tissues) among various breeds.

## 2 Materials and methods

### 2.1 Chicken breeds and sampling

In this investigation, eight chicken breeds and crosses were used (Table 1), which were kept in grower cages and fed following recommendations as prescribed by the Federal Scientific Center “All-Russian Poultry Research and Technological Institute” of the Russian Academy of Sciences (Imangulov et al., 2013). Seventy fertile hatching eggs per breed were used for incubation, while the proper embryo samples were analyzed at embryonic age of 7 (E7) and 14 (E14) days. The content of NO metabolites was determined in embryos at E7, and the DGE level of myogenesis associated genes in the tissues of the breast and thigh muscles was assessed at E14. Incubators Stimul Ink-1000 (OOO Stimul Group, Russia) were used for incubation. Temperature was 37.6°С during the incubation period and 37.2°С at hatching. To obtain homogenates from the whole E7 chick embryos (four per breed), the egg contents were used after removing the eggshell. The contents were processed in a glass homogenizer for 8 min at 40 fpm and 6°C. A tissue grinder was used to obtain breast and thigh muscle tissue homogenates at E14 followed up by total RNA isolation using the RNeasy Midi Kit (QIAGEN, Hilden, Germany) according to the manufacturer’s instructions.

### 2.2 DGE assessment

Relative DGE levels of myogenesis associated genes were examined in the tissues of the breast muscles (Table 2) and thigh muscles (Table 3) in at least five E14 chick embryos per breed (with three technical replicates per sample). Using quantitative real-time PCR and sets of gene-specific primer pairs described elsewhere (e.g., Cazzato et al., 2014; Supplementary Table S1), we analyzed DGE for the following seven genes: *MSTN*, myostatin; *GHR*, growth hormone receptor; *MEF2C*, myocyte enhancer factor 2c; *MYOD1*, myogenic differentiation 1; *MYOG*, myogenin; *MYH1*; and *MYF5*, myogenesis factor 5. For internal DGE control, the TATA-binding protein (*TBP*) gene was used.

**TABLE 2:** Relative DGE levels defined by raw FC values (type I data) in the breast muscle tissues of E14 chick embryos as estimated in the studied breeds.

Breeds	Genes^a^						
	*MSTN*	*GHR*	*MEF2C*	*MYOD1*	*MYOG*	*MYH1*	*MYF5*
Broiler	11.55	6.63	6.59	11.31	7.46	−41,760.00	−7.57
White Cornish	4.89	5.62	2.91	2.19	−4.32	−16.22	−685.02
Plymouth Rock White	6.59	4.35	4.00	2.87	78.25	−24.42	−4.76
Yurlov Crower	121.9	69.1	302.3	−7.11	2.04	1.07	−5.90
Brahma Buff	41.07	31.78	219.8	−25.46	−1.95	−1.73	−8.57
Orloff Mille Fleur	2.41	3.32	2.33	16.11	5.58	−16,270.00	−37.53
Layer	4.72	4.79	4.14	4.59	1.03	−29.45	−66.26
Uzbek Game	1.18	2.51	1.45	−81.01	−106.9	−11,990.00	−4.47

^a^ *MSTN*, myostatin; *GHR*, growth hormone receptor; *MEF2C*, myocyte enhancer factor 2C; *MYOD1*, myogenic differentiation 1; *MYH1*, myosin heavy chain 1; *MYOG*, myogenin; *MYF5*, myogenic factor 5. Internal control gene used: *TBP*, TATA-box binding protein.

**TABLE 3:** Relative DGE levels defined by raw FC values (type I data) in the thigh muscle tissues of E14 chick embryos as estimated in the studied breeds.

Breeds	Genes^a^						
	*MSTN*	*GHR*	*MEF2C*	*MYOD1*	*MYOG*	*MYH1*	*MYF5*
Broiler	3.86	3.07	2.36	18.77	6.73	−10,020.00	−6.45
White Cornish	4.03	3.05	−1.69	−12.13	−4640.29	−335.46	−25,531.63
Plymouth Rock White	4.50	2.95	−1.02	13.18	1.39	−115.36	−33.36
Yurlov Crower	46.53	26.10	494.56	28.44	1.39	2.30	195.36
Brahma Buff	8.86	3.72	63.39	8.78	−2.70	1.37	38.02
Orloff Mille Fleur	−1.28	1.62	1.78	6.06	3.63	−8,481.00	−18.90
Layer	1.25	1.31	2.46	1.08	−78.25	6.23	−87.43
Uzbek Game	3.25	4.92	−4.79	−13.93	−118.60	−17,560.00	−2.43

^a^ *MSTN*, myostatin; *GHR*, growth hormone receptor; *MEF2C*, myocyte enhancer factor 2C; *MYOD1*, myogenic differentiation 1; *MYH1*, myosin heavy chain 1; *MYOG*, myogenin; *MYF5*, myogenic factor 5. Internal control gene used: *TBP*, TATA-box binding protein.

Based on the results of DGE assessment (Tables 2, 3), a Type I dataset was formed, which included raw values of fold change (FC) as a derivative of *C*_T_ (Livak and Schmittgen, 2001; Schmittgen and Livak 2008). If there was upregulated DGE of a gene relative to the internal control gene, i.e., when ∆*C*_T_ < 0, FC value was calculated using the following formula: $FC=2^{{–∆C}_{T}}$ . In the case of downregulated DGE of a gene relative to the internal control gene, i.e., when ∆*C*_T_ > 0, FC was determined using the following formula: $FC=\frac{–1}{2^{–∆C_{T}}}$ (Schmittgen and Livak 2008). Subsequently, four datasets were used for DGE assessment, including one raw FC dataset (I) and three transformed datasets (II to IV). Herewith, appropriate normalizing algorithms were applied for calculating values of shifted DGE levels relative to each other, so that the numbers resulted from mathematical transformation (normalization) were more adequate and convenient for further mathematical processing and analyses (see Supplementary Information SI1 for further details).

### 2.3 Estimation of embryonic NO oxidation rate

The content of NO metabolites in the E7 embryo samples was tested no later than 30 min after sampling. We used the enzymatic sensor we previously developed (Titov, 2011; Titov et al., 2016). Its detecting sensitivity is based on property of nitrite, nitrosamines (RNNO), RSNO, DNIC, and RNO_2_ to inhibit catalase in the presence of halide ions and on their loss of this property under the influence of factors different for each group of compounds. The nitrate content was estimated after reduction with vanadium trichloride to nitrite followed by quantitative determination (Titov, 2011). The enzymatic sensor is designed using a highly sensitive calorimeter Dithermanal (Vaskut-EMG, Hungary). Since the catalase process is highly exothermic (47.2 kcal/1 mol of released oxygen), its kinetics can be monitored by the kinetics of heat production accompanying this process (Titov, 2011; Titov et al., 2016). This method enables estimating the content of NO derivatives without preliminary sample preparation, since there is no need to remove colored impurities and turbidity in samples. The sensor sensitivity is up to 50 nM (Titov, 2011; Titov et al., 2016). The classical Griess reaction method was also used to determine nitrite (Tarpey et al., 2004). DNIC containing two glutathione (GSH) molecules was used as NOD according to the technique we previously described (Titov, 2011; Titov et al., 2016). Solutions prepared in sterile saline were administered *in ovo* before incubation using injection into the air cell of the egg.

### 2.4 Analysis of embryonic development and postnatal growth

For a comparative assessment of the features of embryonic development and postnatal growth in chicks of various breeds, the following indicators were measured: mean weight of fertile eggs prior to incubation, body weight (BW) of chicks at three ages (1, 14, and 28 days) and the degree of NO oxidation to nitrate in the homogenates of E7 embryos. To expand the representative set of various breed types, the following breeds/crosses were also added to the eight initial breeds: two BR crosses, Cobb 500 (BRC) and Ross 308 (BRR); one game breed, Malay Game; and two dual purpose breeds, Andalusian Blue and Blue Meat-Egg Type (BMET) that was selected from the Andalusian Blue breed. A total of 13 chicken breeds were used within this research phase (Supplementary Table S2). Postnatal growth rate (GR) due to the growth of skeleton and muscles, primarily the breast and thighs, was estimated by the degree of BW gain over the first 2 and 4 weeks of life. Accordingly, GR was calculated for 2 weeks (GR2wk) and 4 weeks (GR4wk) by dividing the respective values of BW at 2 and 4 weeks by BW at day old. Further, we also tested relationship between DGE levels of myogenesis associated genes assessed in E14 embryos and GR2wk/GR4wk values.

### 2.5 Principal component analysis, clustering, and statistical processing

Principal component analysis (PCA) and PCA plotting were performed in RStudio (version 1.1.453; RStudio Team, 2016) using the ggplot2 library (version 3.3.5; Wickham, 2009; Pedersen, 2021; Wickham et al., 2021). In addition, PCA plots were built using the web toolbox ClustVis (Metsalu and Vilo, 2015) designed for visualizing clustering of multivariate data. PCA plots were originally obtained by applying the unit variance scaling to rows of the original Type I raw data matrix (with preserving their signs). To calculate principal components, multilevel singular value decomposition with imputation was used. Heat maps and their accompanying clustering trees were built using ClustVis and Euclidean distances for both rows and columns of the matrix (with the average option selected for the linkage method). Additionally, PCA and hierarchical clustering procedures were employed using the Phantasus web application (Zenkova et al., 2018).

Hierarchical clustering was also performed using the pvclust package in R (Suzuki and Shimodaira, 2006). For clustering, the Unweighted Pair-Group Method Using Arithmetic Averages (UPGMA) was applied using the Euclidean distance measure. Bootstrapping with 10,000 iterations was implemented for validation. Using the fviz_nbclust() function from the factoextra package (Kassambara and Mundt, 2017), optimal number of clusters was chosen using the elbow method (Zhao et al., 2008). To test significance of the UPGMA-based hierarchical clustering output, agglomerative coefficient that measures magnitude of the clustering structure found (values close to 1 suggest a strong clustering structure) was calculated using the “agnes” function from the “cluster” package (version 2.1.2; Maechler et al., 2021). Trees of phylogenetic relationships between breeds were constructed using the Neighbor Joining method (Saitou and Nei, 1987) using the online T-REX tool (Boc et al., 2012). Two options were used to build trees: radial topology 1) with proportional lengths of edges and 2) without it.

Manipulations of normal mathematical processing of primary data were carried out using MS Excel. In addition, BioStat software package and RStudio (version 1.1.453; RStudio Team, 2016) were also used for statistical analyses. To assess distribution normality of quantitative traits, the Shapiro–Wilk test was applied using the base function shapiro.test() for R. Since the data did not have a normal distribution, correlation analysis was performed using the Spearman’s rank-order correlation test and the base function cor() for R. Data visualization was performed using the corrplot package (version 0.90) for R (Wei and Simko, 2021).

## 3 Results

### 3.1 Analysis of DGE patterns of myogenesis associated genes in different chicken breeds

#### 3.1.1 Relative DGE data

As follows from the Type I data for the relative DGE in muscle tissues of E14 embryos (Tables 2, 3), essential (i.e., two-fold and higher) upregulation and downregulation of the seven myogenesis associated genes studied were observed in various breeds. For example, WC had markedly lower FC values for the *MYF5* gene in both breast and thigh muscles. Considering raw Type I data (Tables 2, 3), one cannot directly identify any apparent pattern in the DGE levels among the breeds studied. At first glance, each breed was characterized by its own combination of up and downregulation of certain genes.

#### 3.1.2 Cluster analysis of DGE patterns

**FIGURE 1:**
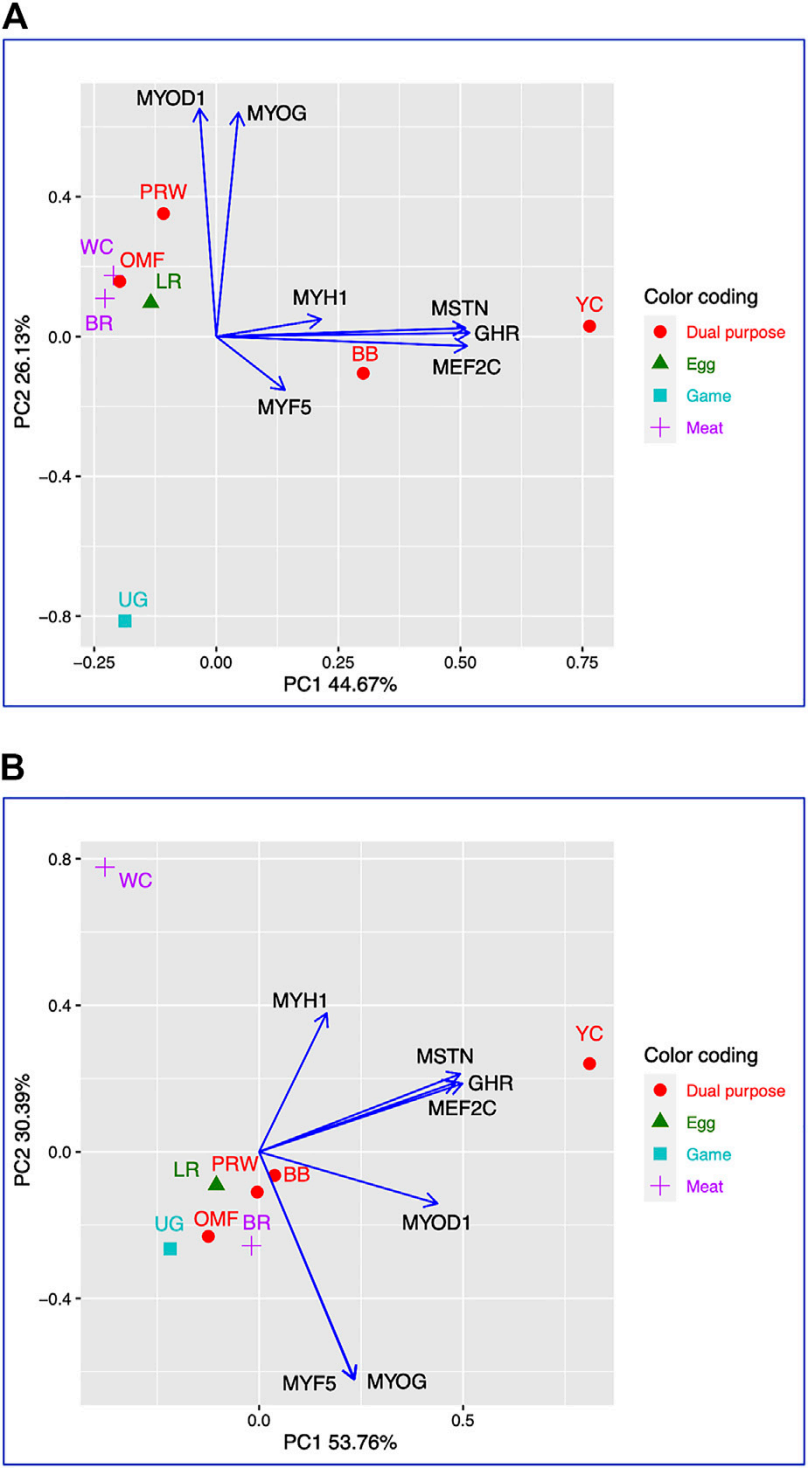
PCA plots generated using the ggplot2 library for the DGE Type I data for the seven genes in the breast **(A)** and thigh **(B)** muscles in the eight breeds studied. *X* and *Y*-axes show principal component 1 (PC1) and principal component 2 (PC2) that explain 44.7% and 26.1% **(A)**, and 53.8% and 30.4% **(B)** of the total variance, respectively. *N* = 8 data points (breeds).

Using for analysis the available raw Type I data matrices for DGE obtained for the seven genes in the breast (Table 2) and thigh (Table 3) muscles in the eight breeds, the clustering structure of the differentially expressed genes (DEG) was analyzed in more detail (Figure 1). Thereby, PCA plots built in R environment using the ggplot2 library demonstrated the DEG clustering patterns for the breast (Figure 1A) and thigh (Figure 1B) muscles, suggesting, in a first approximation, the effects and interactions of DEG in vector form. Gene vectors in the PCA plots (Figure 1) suggested that three genes (*MSTN*, *GHR*, and *MEFC2*) were expressed as if by one “accord” (complex), i.e., interconnected and in one direction, both in the breast (Figure 1A) and thigh (Figure 1B) muscles. In other words, it seems that the *GHR* gene was one of the key ones and was associated with other genes forming a complex. There were also associated and unidirectional vectors for the *MYOG* and *MYOD1* genes expressed in the breast muscles (Figure 1A) and the *MYOG* and *MYF5* genes in the thigh muscles (Figure 1B). The identified “accord” DGE patterns during early muscle development were mainly confirmed when using other variants of PCA analysis and hierarchical clustering (see Supplementary Information SI2 for further details).

#### 3.1.3 Cluster analysis of the studied breeds based on DGE data

**FIGURE 2:**
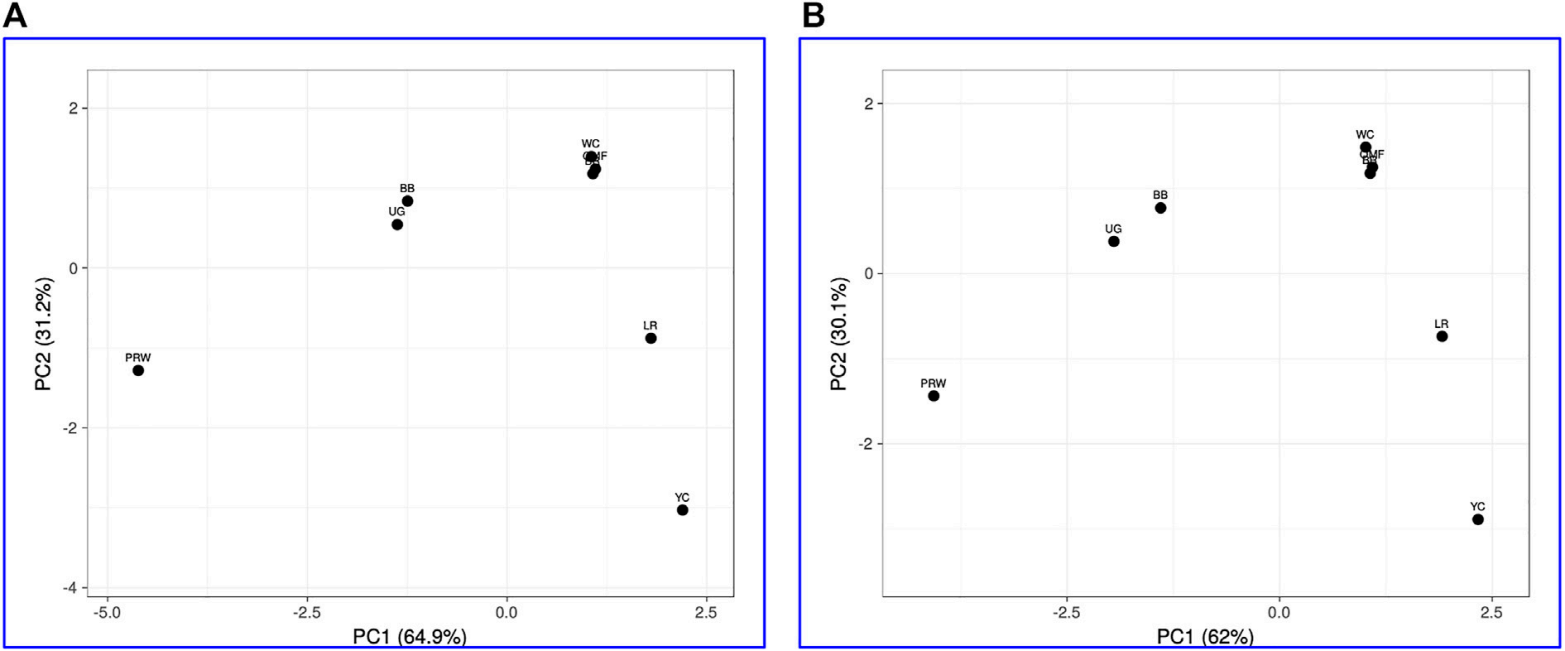
PCA plots generated using the ClustVis tool (Metsalu and Vilo, 2015) and the Type IIIa **(A)** and IIIb **(B)** datasets as inferred for the eight studied breeds and seven tested myogenesis associated genes expressed in the breast muscles. *X* and *Y*-axes show principal component 1 (PC1) and principal component 2 (PC2) that explain 64.9% and 31.2% **(A)**, and 62.0% and 30.1% **(B)** of the total variance, respectively. *N* = 8 data points (breeds).

When analyzing distribution of the eight breeds studied based on DGE data in the breast (Table 2) and the thigh (Table 3) muscles, meaningful clustering patterns were obtained, especially after transformation of primary raw data as outlined below (see also Supplementary Information SI3 for further details). In particular, when using transformed DGE matrices to build PCA plots using the ClustVis web service, similar clustering patterns were obtained based on both Type IIIa (Figure 2A) and Type IIIb (Figure 2B) datasets for the breast muscles. Unlike the raw data I based clustering pattern (Figure 1A), the egg-type LR breed was significantly moved out of the crowded core of breeds in these PCA plots (Figures 2A, B). In general, the PCA results inferred using ClustVis repeated the Neighbor Joining analysis outputs (see Supplementary Information SI3; Supplementary Figure SI3-4).

**FIGURE 3:**
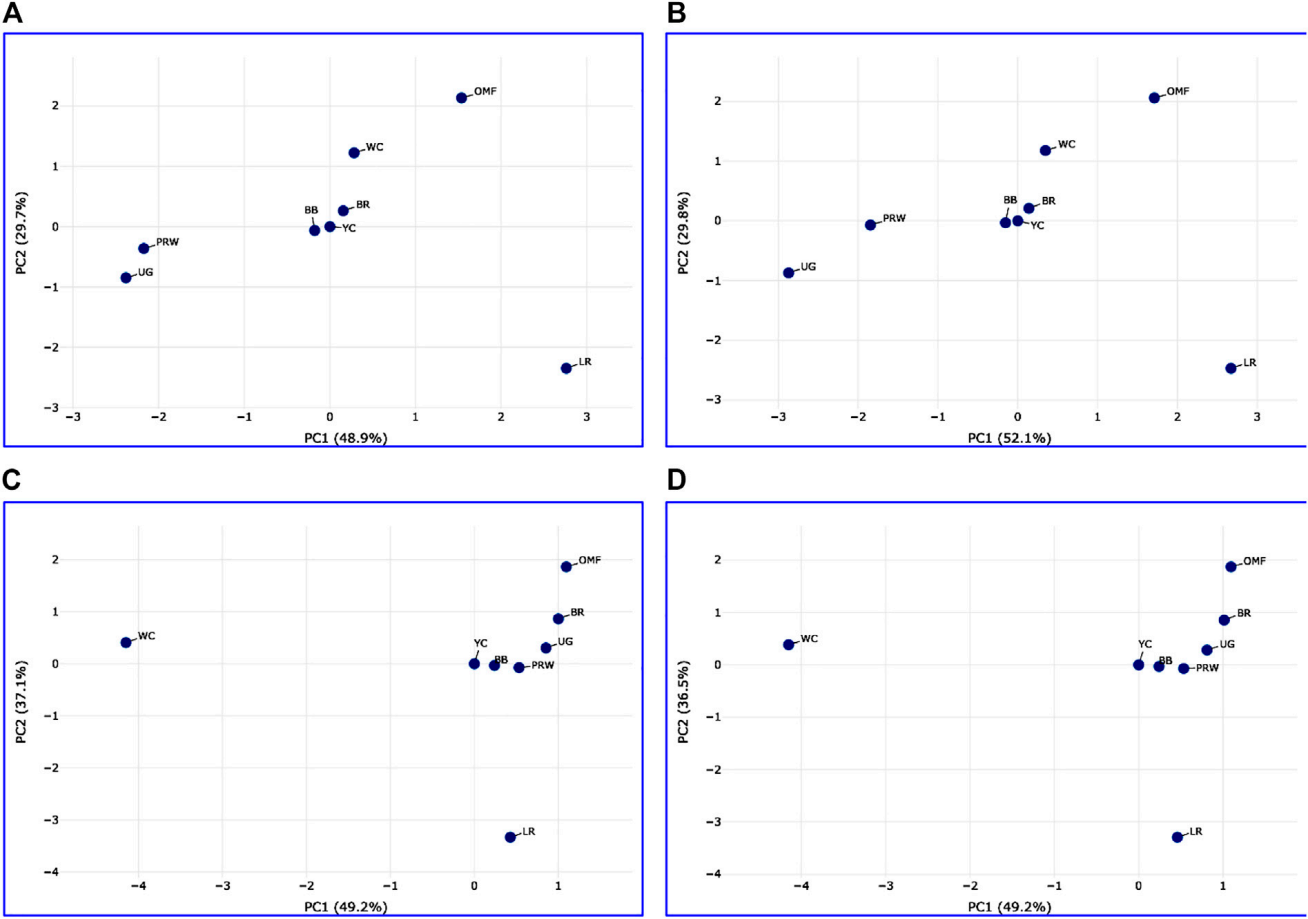
PCA plots generated using the Phantasus tool (Zenkova et al., 2018) and the data Type IIIa **(A,C)** and IIIb **(B,D)** as inferred for the eight studied breeds and seven tested myogenesis associated genes expressed in the breast **(A,B)** and thigh **(C,D)** muscles. *X* and *Y*-axes show principal component 1 (PC1) and principal component 2 (PC2) that explain respective percentage values of the total variance. *N* = 8 data points (breeds).

**FIGURE 4:**
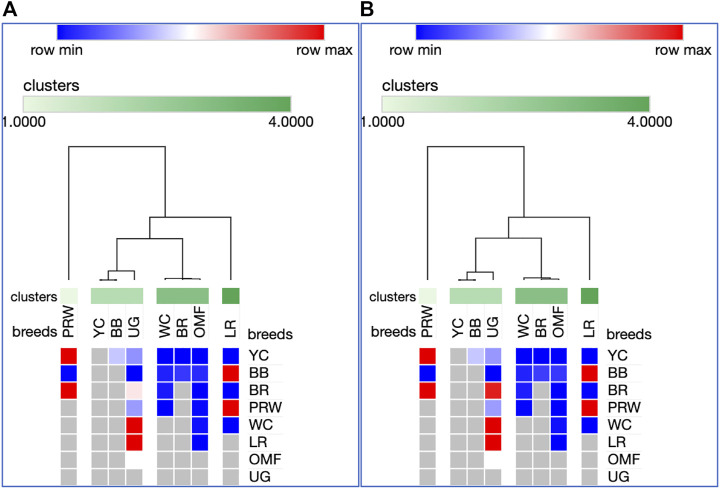
Heatmaps and hierarchical clustering trees based on Euclidean distance metric (with the average option selected for the linkage method) and using the Type IIIa **(A)** and IIIb **(B)** data as inferred for the eight studied breeds and seven tested myogenesis associated genes expressed in the breast muscles.

**FIGURE 5:**
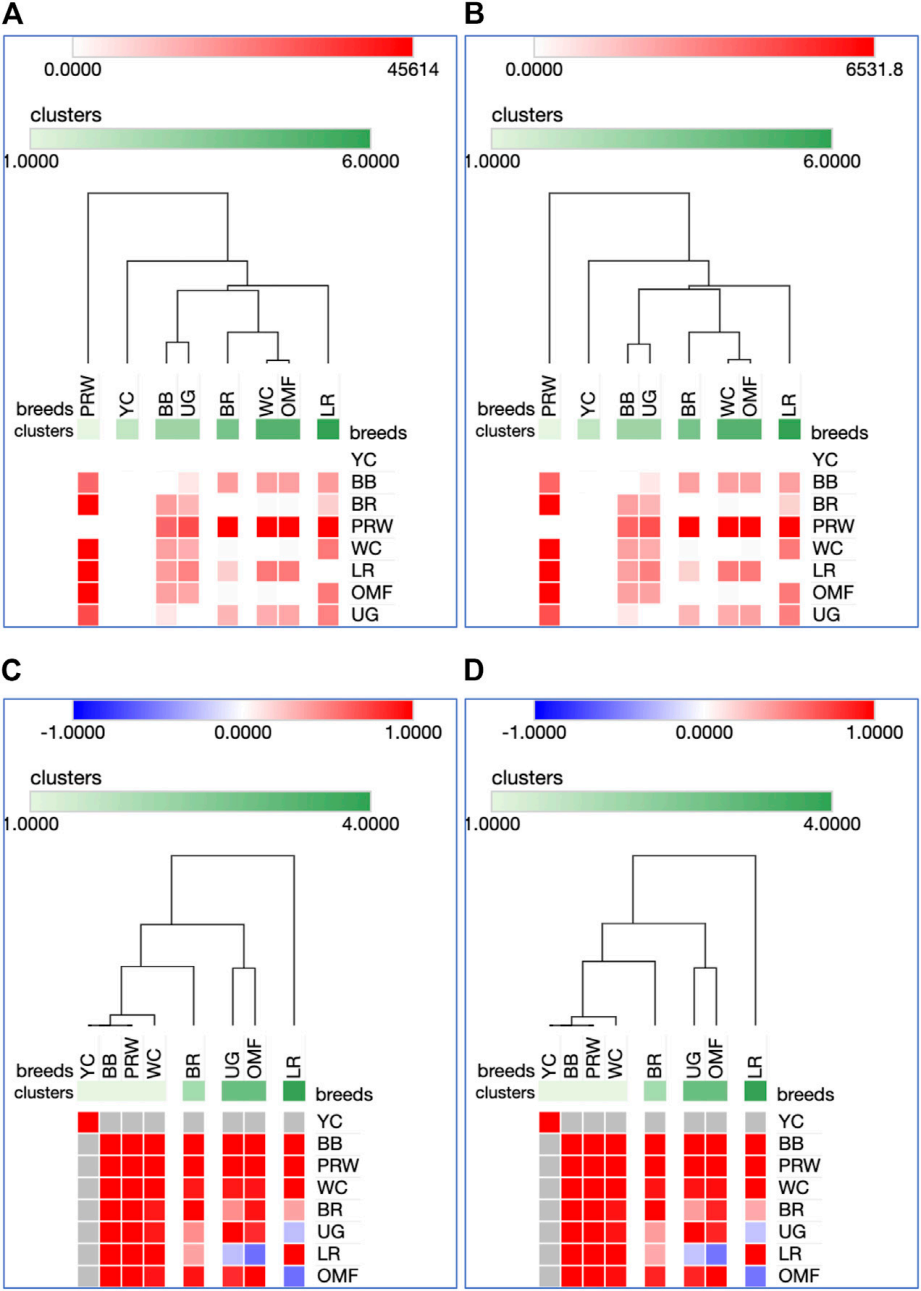
Heatmaps and hierarchical clustering trees based on Euclidean distance metric (with the average option selected for the linkage method) and using the Type IIIa **(A,C)** and IIIb **(B,D)** data as inferred for the eight studied breeds and seven tested myogenesis associated genes expressed in the breast muscles. For constructing the trees, precomputed similarity matrices were built using Euclidean distance **(A,B)** and Pearson correlation **(C,D)** as metrics.

Analysis of the transformed data (i.e., two matrices for the Type IIIa and Type IIIb datasets) was further performed using the Phantasus web service (Zenkova et al., 2018) and the standard PCA algorithm (Figure 3). Additionally, we tested slightly changed the Type IIIa and IIIb datasets through obtaining similarity matrices based on the Euclidean metric (Supplementary Figure S1). The obtained PCA plots for the breast muscles (Figures 3A, B; Supplementary Figures S1A, B) basically repeated the patterns of PCA clustering (Figure 2) obtained using ClustVis (Metsalu and Vilo, 2015). For example, LR again turned out to be distanced from the crowded core of other breeds with a shift to the right plot side. Moreover, the crowded core itself in Supplementary Figures S1A, B was represented by two breeds, WC and OMF, and the BR breed moved away from it to the left. It seems that these PCA plots more plausibly described the DGE results obtained for early myogenesis associated genes in the breast muscles in various chicken breeds studied. In the case of the thigh muscles (Figures 3C, D; Supplementary Figures S1C, D), we had crowded cores of six different breeds with two other breeds being remote from them. At the same time, the apartness of the meat-type (WC) and egg-type (LR) breeds in Figures 3C, D looked most plausible. Note also that there was practically no difference in PCA patterns between the two data types, IIIa (Figures 3A, B; Supplementary Figures S1A, B) and IIIb (Figures 3C, D; Supplementary Figures S1C, D). Overall, the obtained PCA plots (Figure 3; Supplementary Material S1; cf. also the increased sums of the proportions of PC1 and PC2 for the breast and thigh muscles as compared to those for PCA using raw Type I data in Supplementary Information S4) indicated a slightly better resolution of the PCA method in comparison with the Neighbor Joining analysis results (see Supplementary Information SI3; Supplementary Figure SI3-4). Next, another Phantasus option was used, i.e., hierarchical clustering based on the Euclidean metric (Figure 4). In this case, two data types, IIIa (Figure 4A) and IIIb (Figure 4B), were also compared and the resulting trees were identical to each other. This means that both approaches to the transformation of raw data did not contradict each other. According to the character of DGE in the breast muscles, the examined breeds were divided into four main clusters: 1) two meat-type breeds (WC and BR) and one dual purpose breed (OMF); 2) two dual purpose breeds (YC and BB) and one related (by descent) game breed UG; 3) the egg-type LR breed; and 4) the dual purpose PRW breed (female grandparent stock of the BR cross). To improve the sensitivity of hierarchical clustering, both datasets, IIIa and IIIb, were also preliminarily subjected to the procedure for constructing similarity matrices using the Euclidean distance metrics (Figures 5A, B) and Pearson’s correlation coefficient (Figures 5C, D). The generated clustering patterns for Type IIIa data (Figures 5A, C) were respectively identical to those for Type IIIb counterparts (Figures 5B, D).

For DEG in the breast muscles, five main clusters were identified (Figures 5A, B): 1) the one coinciding with the first cluster in Figure 4 and splitting into two subclusters BR and WC + OMF; 2) the one combining BB and UG; 3) LR; 4) YC; and 5) PRW. For the thigh muscles, we had a completely different pattern of hierarchical clustering: 1) one large cluster with two dual purpose breeds, YC and BB, and two BR grandparent stocks, WC and PRW; 2) the meat-type BR breed; 3) the game UG breed and OMF considered as a “semi-game” breed; and 4) the egg-type LR breed. The clustering trees obtained for the breast and thigh muscles (Figure 5) were even more demonstrative than the PCA patterns (Figures 2, 3; Supplementary Material S1). Moreover, these trees showed phylogenetic relationships much better than the Neighbor Joining trees (see Supplementary Information SI3; Supplementary Figure SI3-4).

On the whole, the results of analyzes using PCA and hierarchical clustering suggest the peculiar nature of DGE patterns in both breast and thigh muscles in embryos of two breeds, imported egg-type LR and domestic dual purpose YC, which distinguished these breeds from the rest. At the same time, the proximity of YC to another dual purpose breed, BB, was also noted. In the breast muscles, a distinct DGE pattern was observed in the meat-type BR breed, and it was close to the patterns in meat WC and dual purpose OMF. The dual purpose PRW breed (female grandparent of the BR cross) was also distinguished by the DGE peculiarity in this tissue. We can also suggest the similarity of DGE patterns in the breast muscles in game UG and dual purpose BB. In addition, in the thigh muscles, we also observed a peculiar DGE pattern in meat WC (male grandparent of the BR cross).

### 3.2 Analysis of embryonic development and postnatal growth in various chicken breeds

Indicators of the mean egg weight (EW), BW of chicks at three ages (1, 14, and 28 days), and the degree of NO oxidation to nitrate in the homogenates of E7 embryos for the 13 chicken breeds are presented in Supplementary Table S2. Significant interbreed variability in values of the studied traits was noted.

#### 3.2.1 Early growth traits

Based on the growth data obtained (Supplementary Table S2), it was feasible to identify breeds with approximately similar GR. For instance, breeds such as BR cross Smena 8 (BRS), WC and PRW, or a pair of dual purpose breeds, YC and BB had similar values of BW at 1-, 14- and 28-days of age, respectively. Overall, when assessing the GR values, the three BR crosses, BRS, BRC and BRR, as well as their grandparent stocks, WC and PRW, were expectedly similar. Their GR2wk values ranged from 5.70 to 7.87. The same indicators in game breeds, MG and UG, were 2.24 and 2.61, respectively, in dual purpose breeds they ranged from 2.01 to 2.81, and in LR it was the lowest (1.88). The GR4wk values were again maximum in BR breeds and their grandparent stocks (23.57–26.41), whereas it was 5.42 and 5.67 in game breeds, 4.59 to 6.33 in dual purpose breeds, and 5.25 in LR.

**FIGURE 6:**
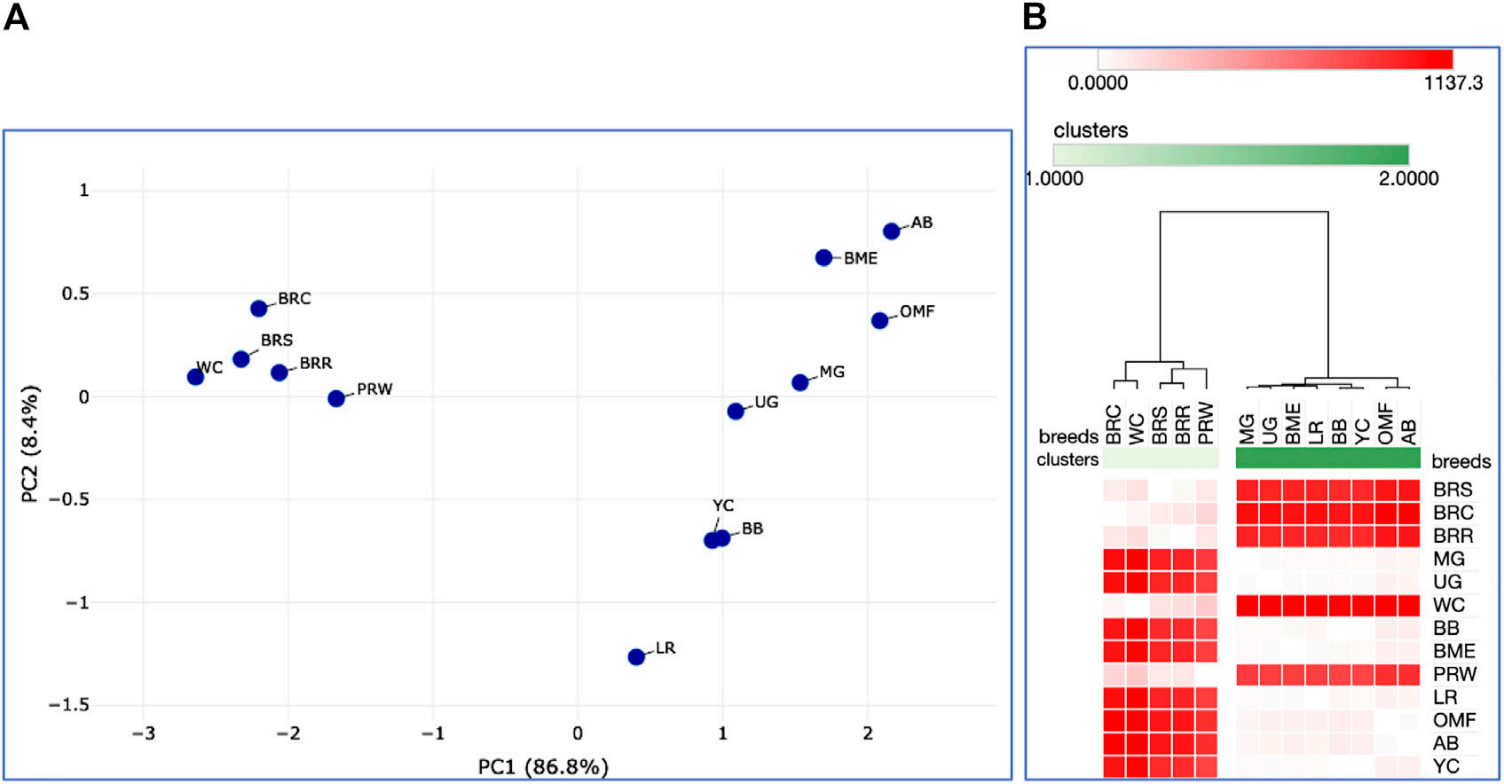
Analysis of the distribution of 13 breeds by early growth traits, including EW and BW of chicks at three ages, as generated in the Phantasus program (Zenkova et al., 2018). **(A)** PCA plot. *X* and *Y*-axes show principal component 1 (PC1) and principal component 2 (PC2) that explain 86.8% and 8.4% of the total variance, respectively. *N* = 13 data points (breeds). **(B)** Heatmap and hierarchical clustering tree using Euclidean distance-based similarity matrix. For clustering, matrix values (for a precomputed distance matrix) were applied as metrics (with the average linkage method option selected).

Further, we deduced and explored patterns of the embryonic and postnatal development in various chicken breeds using PCA and hierarchical clustering analyzes. Based on the indicators of EW (i.e., at the initial point of embryo development) and BW of chicks at three ages (i.e., at three temporal points of post-hatch development), plots in Figures 6A, B suggested the formation of two large clusters occupying respectively, the left and right parts of both graphs: one was a BR cluster (three crosses and two grandparent stocks), and the other consisted of the rest of the breeds. As demonstrated in the PCA plot (Figure 6A), the egg-type LR breed occupied a somewhat remote position at the bottom of the plot. Interestingly, a closely related pair of two dual purpose breeds, YC and BB, formed a separate cluster on the right side, as was also seen for DGE of embryonic myogenesis associated genes (see Figures 3, 4, 5C, D; Supplementary Information SI2; Supplementary Figures SI2-1C, D; Supplementary Information SI3; Supplementary Figures SI3-2, 3, 5C, D). A close pair was also made up by the two game breeds, MG and UG. In addition, in the upper right corner of the PCA plot (Figure 6A), two closely related dual purpose breeds, AB and BME, were located next to each other. In many respects, a similar pattern of breed clustering was observed on the corresponding heat maps (Figure 6A; Supplementary Material S2). Almost the same distribution patterns of the 13 breeds were obtained by adding GR indices to the set of analyzed growth traits (Supplementary Figure S3).

#### 3.2.2 NO exchange in relation to early chick growth

When considering the data on the degree of NO oxidation to nitrate in homogenates of E7 embryos in the 13 chicken breeds (Supplementary Table S2, with the breeds being sorted in descending order by the level of NO oxidation), it can be seen that the highest NO oxidation values were inherent in three BR crosses (BRS, BRC, and BRR), meat-type WC (male grandparent stock) and two game breeds (MG and UG) and accounted for 96.9%–98.1%. High values of this indicator were also observed in two dual purpose breeds, BME and BB (61.8 and 74.1, respectively). The rest of the breeds had contrastingly low values of the NO oxidation degree (2.6% and below). Next, we tested the relationship between the level of embryonic NO metabolism and early postnatal growth of chickens. To do this, the clustering patterns of breeds were checked altogether for traits of NO oxidation at E7 and BW at day old (Supplementary Figure S4). The formed three clusters conformed to the same differences revealed by the level of NO oxidation (Supplementary Table S2), which was understandable since the day-old chicks did not differ much from each other in BW.

**FIGURE 7:**
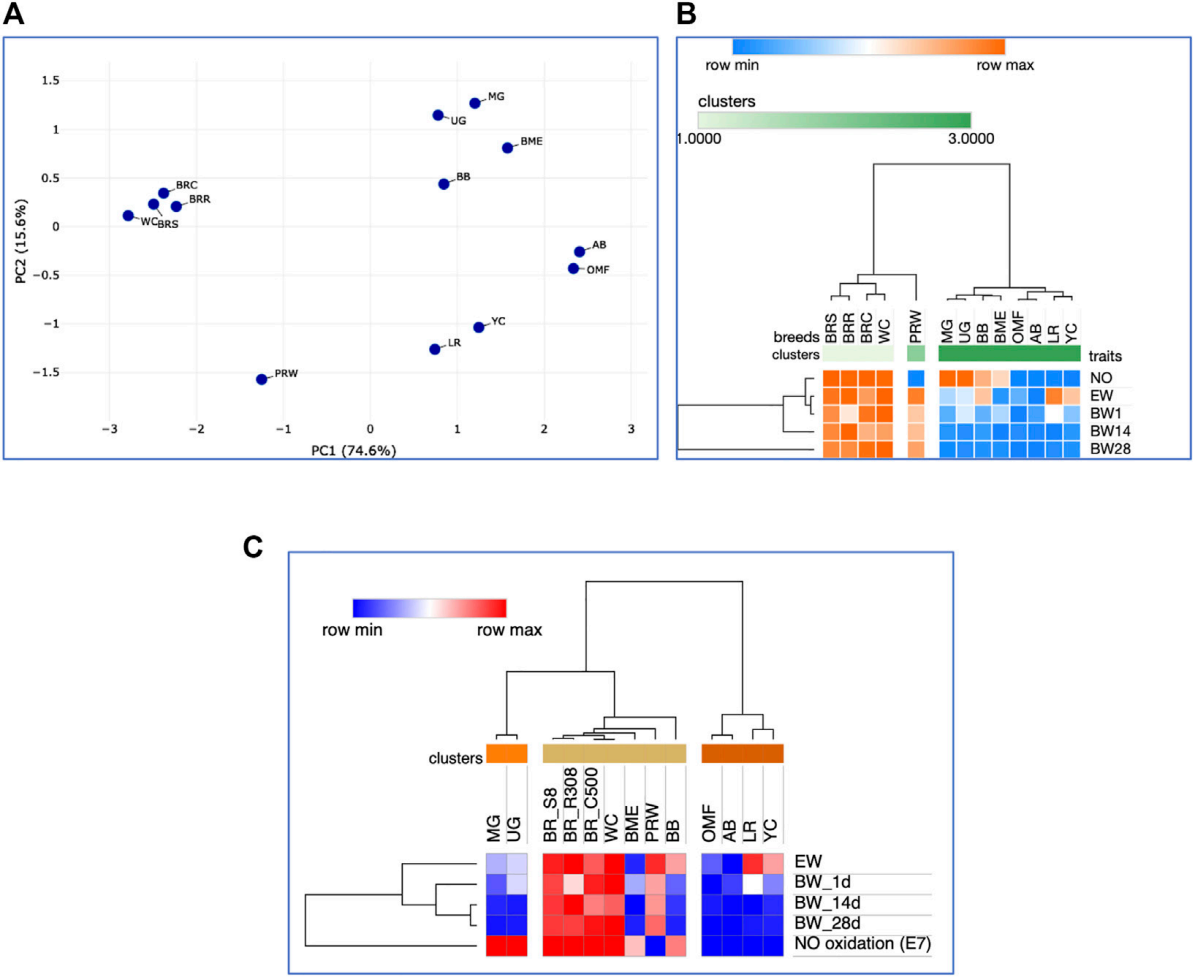
Analysis of the distribution of 13 the breeds by traits of E7 NO oxidation and postnatal growth, including EW and BW of chicks at three ages, performed in the Phantasus program (Zenkova et al., 2018). **(A)** PCA plot. *X* and *Y*-axes show principal component 1 (PC1) and principal component 2 (PC2) that explain 74.6% and 15.6% of the total variance, respectively. *N* = 13 data points (breeds). **(B)** Heatmap and hierarchical clustering tree based on Euclidean distance metric (with the average option selected for the linkage method). **(C)** Heatmap and hierarchical clustering tree using one minus Pearson’s correlation metric (with the average option as linkage method).

As the chicks grew, there were changes in interbreed differences. At 14 days of age, somewhat different clustering patterns were already observed (Supplementary Figures S5, S6). In particular, a pair of game breeds, MG and UG, and two dual purpose breeds, BME and BB, moved away from the BR cluster. The breeds seen in the right cluster in Supplementary Figure S4 formed a crowding pattern, with PRW detached from them and moved closer to the BR cluster. The same patterns of interbreed differences can be generally noted for the entire observation period, i.e., up to 28 days of age of the chicks (Figures 7A-C; Supplementary Material S7). When modifying the hierarchical clustering by applying the One minus Pearson’s correlation metric (using the average linkage method; Figure 7C), the chicken breeds under consideration were divided into three clusters according to their utility purpose: 1) game (MG and UG); 2) meat-type (BRS, BRR, BRC, WC, PRW plus two dual purpose breeds, BB and BME); and 3) the remaining dual purpose breeds and the egg-type LR breed. Notably, when using this option of hierarchical clustering, we obtained a slightly different pattern of effects of the studied traits of early development and growth (Figure 7C): the indicators of the weight of fertile eggs and BW at 1, 14, and 28 days of life were isolated into a separate cluster detached from the E7 NO oxidation index (as also seen in the plot of Supplementary Figure S7).

The data used for analyzing the same five traits (including the E7 NO oxidation) correlated well with each other using the Spearman’s correlation coefficient (Supplementary Table S3; Supplementary Figure S8). The E7 NO oxidation had a significant and positive correlation with the indicators of the BW of chickens, starting from hatching and up to 28 days of age. By adding two more parameters to this set of traits, GR2wk and GR4wk, more differentiated patterns of relationships between breeds were obtained (Supplementary Figures S9, S10). Remarkably, if we look at the relationships of all seven studied traits of embryonic NO metabolism and early growth of chicks (Supplementary Figures S9B, S11), we can see that GR2wk and GR4wk almost coincide with each other, suggesting their almost equal contribution to the observed clustering patterns of the 13 breeds. EW and BW of day-old chicks were close to each other that can be indicative of insignificant interbreed differences in these indicators of the earliest chicken development. Further, this cluster in Supplementary Figure S9B joined with the E7 NO oxidation index, which is also understandable, since these three traits characterize embryonic development in the studied breeds. BW at 14 and especially at 28 days of age made a much stronger contribution to the pattern of interbreed differences, outlining specific trajectories in the further development and growth of birds of a particular breed.

#### 3.2.3 Unified early development and growth model

We also tested a model embracing all various traits studied, i.e., DGE of myogenesis associated genes, NO metabolism in embryos, as well as seven indicators of early chick growth in the eight breeds. At first, using DGE indices for the seven genes, i.e., *MSTN*, *GHR*, *MEF2C*, *MYOD1*, *MYOG*, *MYH1*, and *MYF5*, in the tissues of the breast (Table 2) and thigh muscles (Table 3) in E14 chick embryos, the respective pairwise Spearman’s correlation coefficients were calculated (Supplementary Tables S4, S5; Supplementary Figure S12). As a result of this analysis, significant pairwise correlation values were found between DGE indicators of some genes and between growth indicators. In particular, a significant correlation was confirmed between DGE levels for the three genes *GHR*, *MSTN*, and *MEF2C* in the breast muscles (Supplementary Table S4; Supplementary Figure S12A). This may reflect their key role in the embryonic myogenesis of the breast muscles in all the examined chicken breeds. In the thigh muscles, we have other pairs of significantly correlated genes: *GHR*–*MYF5*, *MEF2C*–*MYH1*, and *MYOG*–*MYF5*, suggesting clear differences in the myogenesis of different chick embryo muscle tissues. In terms of early growth and chick BW changes (Supplementary Tables S4, S5; Supplementary Figure S12), significant correlations were found for the two earlier measures (pre-incubation EW and post-hatch BW) as well as for the three postnatal BW measures (at 1, 14, and 28 days of life).

When considering the results of hierarchical breed clustering within this model, the broiler breeds BR and PRW constituted a close cluster, while another broiler breed, WC, was located to the side in all PCA plots and trees (Supplementary Figures S13, S14). The second large cluster consisted of egg-type LR, game UG and all dual purpose breeds, with one of the members of this cluster, YC, being located remotely from it. It is also worth noting that the preliminary transformation of the dataset into the Euclidean distance-based similarity matrix (similar to obtaining the Type IIIa data) reflected this clustering pattern even more clearly (Supplementary Figures S14B, C): WC stood away even further from BR and PRW, and the second cluster turned out to be very compact and crowded, with a very small separation of YC from it. In addition, judging from the trait clustering pattern (Supplementary Figure S13B), the used set of traits was clearly divided into two large clusters in terms of its contribution to interbreed differences. The first cluster included all DGE indicators (except for the correlated *MYOG* and *MYOD1* in the breast muscles), while the characteristics of early development and growth were in the second cluster.

**FIGURE 8:**
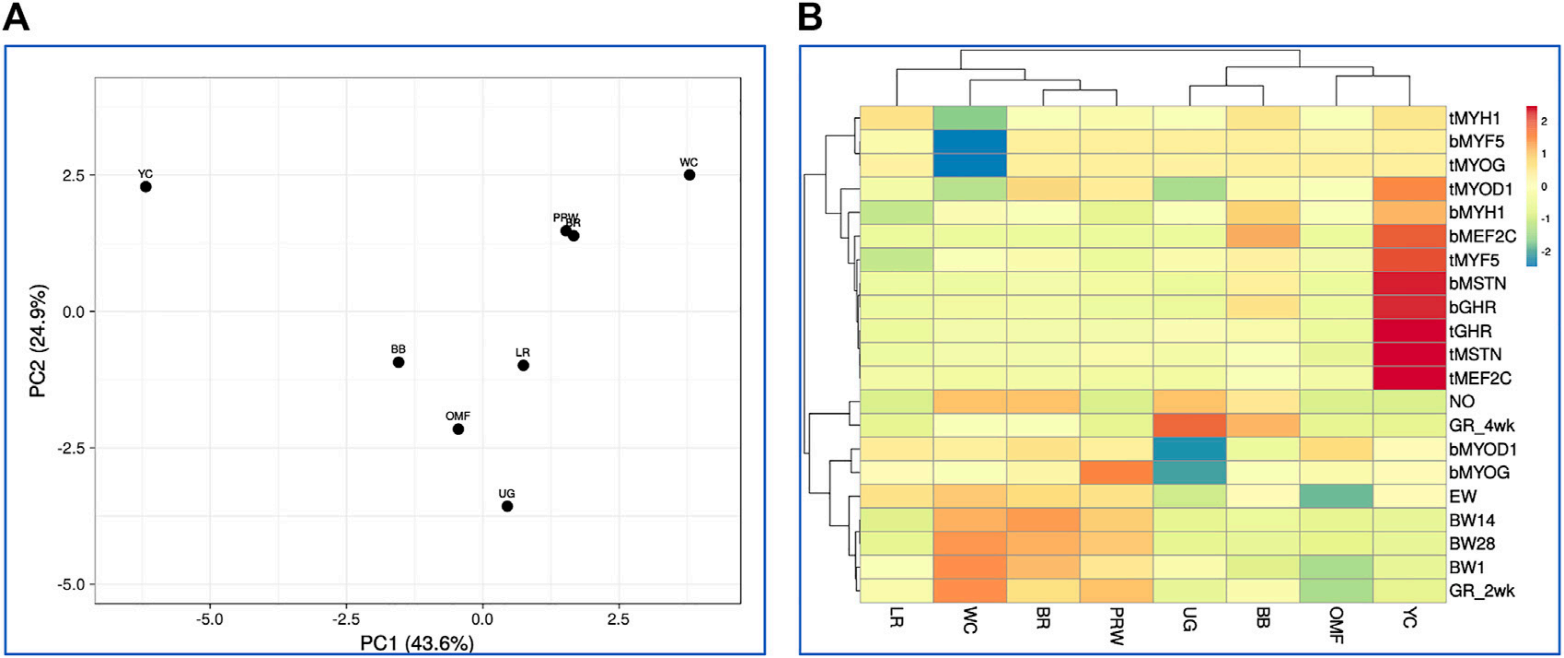
Distribution of the eight breeds based on the analysis of relationships between 21 traits (DGE of myogenesis associated genes and NO metabolism in embryos, as well as indicators of early chick growth) as performed in the ClustVis program (Metsalu and Vilo, 2015). **(A)** PCA plot. Unit variance scaling was applied to rows; singular value decomposition with imputation was used to calculate principal components. *X* and *Y*-axes show principal component 1 (PC1) and principal component 2 (PC2) that explain 43.6% and 24.9% of the total variance, respectively. *N* = 8 data points (breeds). **(B)** Heatmap and clustering trees using Euclidean distances (with the average option selected as linkage method).

Finally, based on this model that combines all the 21 traits studied in the eight breeds (including GR2wk and GR4wk), we observed again similar patterns in the clustering of breeds and features (Figure 8; Supplementary Figure S15). On the right side of the PCA plot for the distribution of the entire set of 21 traits accounted for in the eight breeds (Supplementary Figure S16), one can observe the main crowded core of DGE measures with several outliers, for example, *MEF2C* and *MYF5* (in the breast and thigh muscles), and *MYOG* (in the thigh muscles). From this core to the left side of the PCA graph, indicators of development and growth lined up almost along the same vector with increasing distance from NO and EW to BW28. Almost the same division into two large groups of features was noted on the hierarchical clustering plot in Figure 8.

**FIGURE 9:**
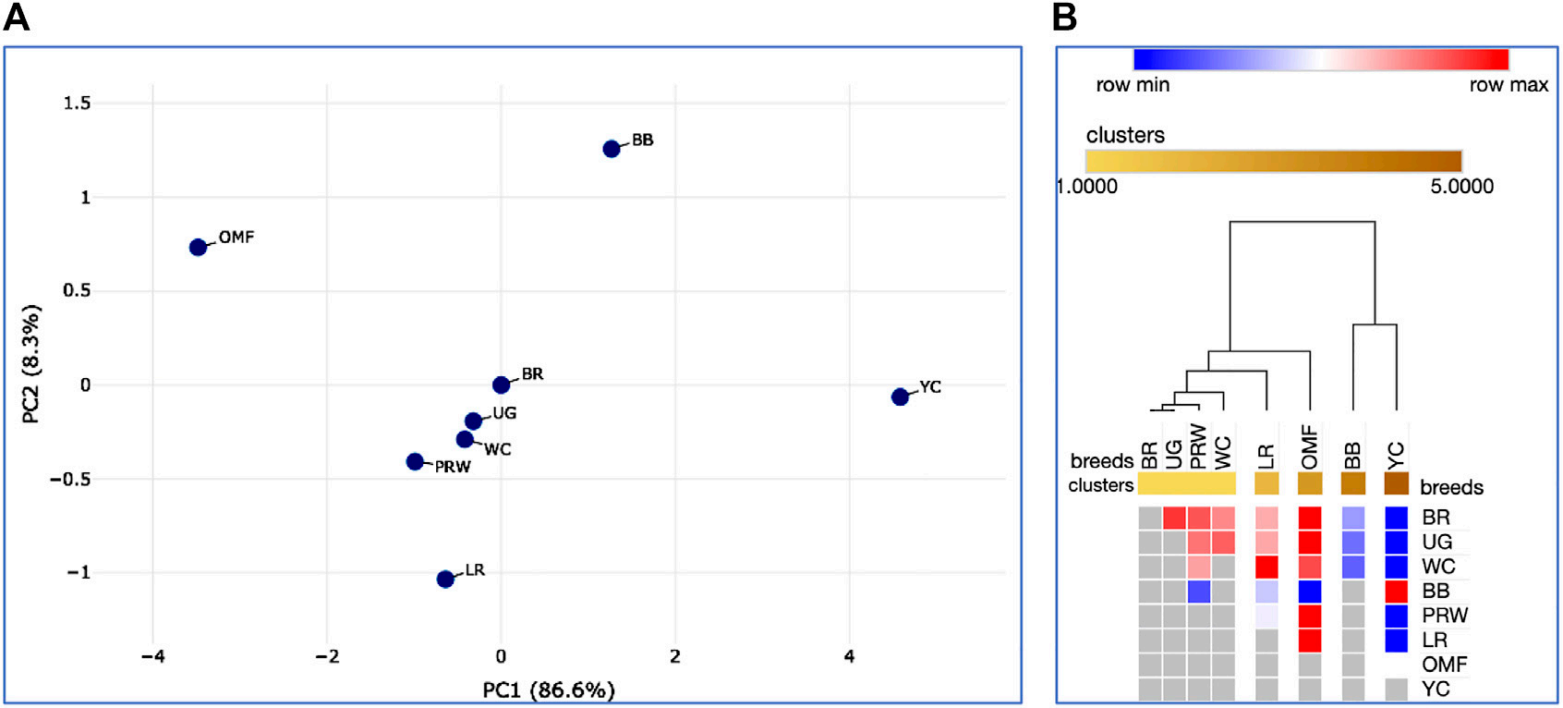
Analysis of the distribution of the eight breeds for 21 traits (DGE of myogenesis associated genes and NO metabolism in embryos, as well as indicators of early chick growth) performed in the Phantasus program (Zenkova et al., 2018). **(A)** PCA plot. *X* and *Y*-axes show principal component 1 (PC1) and principal component 2 (PC2) that explain 86.6% and 8.3% of the total variance, respectively. *N* = 8 data points (breeds). **(B)** Heatmap and hierarchical clustering tree based on Euclidean distance metric (with the average option as linkage method).

Additionally, we calculated Spearman’s correlation coefficients for pairwise comparison of eight breeds and the same 21 indicators (Supplementary Tables S6, S7; Supplementary Figure S17). Using Spearman’s correlation coefficient data, interbreed clustering patterns were tested on PCA and hierarchical clustering plots (Figure 9). These graphs showed the formation of a central core of breeds, composed of BR and their two parental forms WC and PRW, as well as the game UG. Egg LR, dual purpose OMF, and a subcluster of two dual purpose breeds BB and YC were located at a distance from this core and along differently directed vectors. Significant pairwise Spearman’s correlations supported the previously found relationships between certain indicators of early myogenesis and postnatal growth (Supplementary Table S7; Supplementary Figure S17B). Thus, highly correlated DGE profiles of the *MSTN*, *GHR*, and *MEF2C* genes in the breast muscles were verified. In the thigh muscles, the DEG levels of *GHR* and *MYF5*, as well as *MYOG* and *MYOD1*, were positively correlated. The DGE indices of the *MSTN* gene in the breast and thigh muscles in different breeds had a high and significant correlation; this was also observed in the case of the *MEF2C* and *MYOG* genes. DGE of a few genes in the breast muscles positively correlated with that of other genes in the thigh muscles, e.g., *MYOG* in the breast muscles and *MYOG* in the thigh muscles. At the same time, when comparing DGE in pairs, some other genes negatively correlated with each other, in particular, *MYOD1* in the breast and *GHR* in the thigh muscles. All this contributed to the peculiar DGE profiles observed for the myogenesis associated genes studied. Mutual positive pairwise correlation between the early chick growth indicators (EW, BW1, BW14, BW28, and GR2wk) in various breeds was also confirmed.

## 4 Discussion

### 4.1 Embryogenesis, postnatal growth, and DGE of myogenesis associated genes

The present study suggested consistent DGE patterns of the *GHR* and *MSTN* genes, as well as *MYH1*. In certain breeds, however, myogenesis associated genes worked differently in the thigh muscles than in the breast muscles as evidenced by slightly different breed clustering patterns revealed by PCA and hierarchical clustering. Using various analytical approaches (e.g., Figure 1) these different effects of the myogenesis associated genes’ functioning depending on the type of muscle were also demonstrated. As an example, there were a few reports describing the *MYOG* and *MYF5* genes expressed in concert (Auradé et al., 1994; Conerly et al., 2016), as we observed in the case of correlated DGE of these genes in the thigh muscles.

In the primary processing of DGE data reflecting features of the synthesis of mRNA molecules, it is important to develop solutions for reliable DEG identification. Genes are considered to be differentially expressed if they satisfy the *p*-value test and the FC test. If we take into account that the FC value is understood as a multiplicity factor, operations on it should be performed appropriately. In the present study, just such transformations were carried out. To establish DEG, they were ranked based on their FC (Mutch et al., 2002). A number of generally accepted procedures can be helpful in searching for DEG. When analyzing microarrays or RNA-Seq data for thousands of genes, the first step should include removal (filtering) of genes with a very low number in all libraries. There are both biological and statistical reasons for this (Chen et al., 2016). Thus, truncation, filtering, and transformation of data are primary tools in searching for DEG. The main purpose of these data manipulations is to narrow the search for genes of interest. In the current study only focused on the seven myogenesis associated genes, the task was not so much to search for DEG as there was a common goal—to isolate the characteristic indicators of early myogenesis in various chicken breeds created by divergent selection and belonging to one or another utility type.

As has already been established, embryonic metabolism can be divided into three major phases (Spiridonov et al., 2017). The first phase, or the embryonic period, begins in the oviduct and lasts in chickens up to E8. At this time, temporary embryonic organs are already functioning; nutrients are supplied from the yolk; and breathing occurs through the blood vessels of the yolk sac and, at the end of this phase, additionally through the vessels of the allantois. In the second “pre-fetal” period (from E9 to E14), nutrition occurs from yolk and then intra-intestinally *via* amniotic fluid; respiration—with the help of allantois; excretion of metabolic products through the mesonephros. The third “fetal” period (from E14 to E20) is characterized by the most rapid growth of the permanent organs of the embryo, nutrition with protein dissolved in the amniotic fluid, excretion of uric acid through the metanephros, and allantoic respiration (Spiridonov et al., 2017). The extraction of nutrients from the protein and yolk is largely commensurate with the body growth until the embryo completion by E14. Since E15, the metabolic profile of the embryo muscles changes. From E15 to E19, the chick embryo prepares for hatching by increasing the relative mass of the liver and muscles, by elevating the concentration of protein in the muscles, and by accumulating glucose and glycogen in it (Pulikanti et al., 2010). The hourglass model of embryo development suggests that the middle, or phylotypic, stage of embryonic development, when the body plan characteristic of this type of animal is laid down, have an increased evolutionary conservatism compared to the early and late stages. In addition, it turned out that genes that work at the middle development stages are characterized by increased multifunctionality: Many of them perform various functions at different development stages and in different parts of the body (Irie and Kuratani, 2014; Furusawa and Irie 2020).

Genes that control the middle stages of development are characterized by increased pleiotropy (multifunctionality). Many of them are involved not only in the rapid morphogenetic processes of the phylotypic stage of development, but also in other processes at other stages. Apparently, these genes were involved more often in the course of evolution than others to perform novel functions, e.g., when old regulatory genes can acquire new functions. Multifunctional genes operating at the phylotypic stage are so important for the normal development of an organism, that the system of their DGE regulation gained increased noise resilience in the course of evolution (Hu et al., 2017). Metabolic pathways are dependently linked to each other and share intermediate metabolite substrates, so they require precise homeostatic control. The amount and type of substrates available to the embryo trigger the production of hormones, which in turn control DGE of genes for enzymes that regulate the flows in these pathways. From about E5, the chorioallantoic membrane begins to develop, and from E8 it becomes the main means of oxygen uptake. In this study, we turned our attention to the turning points that occur at E7, when the creation of chorioallantois occurs and there is a change in the process of respiration from limited to high oxygen consumption (Tullett and Deeming, 1982; Reijrink et al., 2008). The second turning point of embryonic development is E14. The breast muscle growth hormone (GH) and its receptor (GHR) in the third period of embryonic development gradually begin to be expressed and reach its peak 48 h before hatching (data obtained in turkeys; de Oliveira et al., 2013). It is known that turkey embryos reach full body size by the pipping stage (3 days before hatching), and an increase in muscle mass is a consequence of increased tissue hydration (Vleck, 1991). *GHR* is one of genes considered when calculating indices for the breast and thigh muscles. As we found, there was a positive correlation between the DGE indices for *GHR*, *MSTN*, and *MEF2C* in the breast muscles at the level of .93 (*p*-value <.001).

In terms of GR patterns, we discovered a relationship between the increase in BW and utility type change from egg to meat type. These data also showed that the game breed, UG, is not characterized by the same body muscularity and GR as compared to the meat breeds, meaning that:

1. Commercial meat-type breeds and BR crosses have the highest rates of BW growth as a result of long-term artificial selection for meat traits.
2. The game breed was not subject to such selection. For any game breed, the most important are fighting qualities with a fairly light BW, which ensures the mobility of the bird in cock fights arranged in the past. Therefore, the BW growth in game chickens is more consistent with that in egg-type breeds.

### 4.2 The mechanism of interrelation of embryonic NO oxidation and post-hatch GR

From the results of our study (Supplementary Table S2), it follows that in E7 embryos (more precisely, in their muscle tissues) in almost all meat breeds and crosses, a high degree of NO oxidation to nitrate takes place. At the same time, NO oxidation to nitrate in embryos of egg-type breeds is minor. Selection for increased meat performance within the same breed (Andalusian Blue) resulted in an elevation in the oxidation degree of NO synthesized during embryogenesis in the BMET breed. Because BMET is a product of the Andalusian Blue chickens selected for meat traits, the degree of NO oxidation in the BMET embryos was ∼62%, while in the Andalusian Blue, like in all egg-type breeds, it was marginal (∼2%; Supplementary Table S2). Also, GR4wk in the BMET breed was significantly higher than that in the Andalusian Blue breed (*p* < .05; Supplementary Table S2).

An even greater difference in GR was observed, for example, for the BRS–LR pair (Supplementary Table S2). Broilers are produced by crossing male and female grandparent stocks, which in turn are also the result of crossing certain lines of WC and PRW breeds, respectively. Supplementary Table S2 shows that the three broiler crosses and their male grandparent stock breed (WC) were characterized by almost complete NO oxidation in the embryos (∼97%–98%). Conversely, in the female grandparent stock breed (PRW), NO is practically not oxidized. All these data, on the one hand, indicate that the oxidation degree of embryonic NO is genetically determined. However, the nature of its inheritance suggests that the intensity of NO oxidation is determined not by any specific gene but, apparently, by the combination of several DEG, although we were unable to detect significant association of NO oxidation with the seven myogenesis associated genes tested. It is also known that the oxidation degree of embryonic NO does not depend on the incubation conditions, as well as the age and maintenance conditions of female breeders (Titov et al., 2012; Titov et al., 2018).

Based on the data obtained, we can hypothesize that NO is involved in specific processes of avian embryogenesis. First of all, the fact that in BR embryos most of the deposited NO (∼90%) is oxidized to nitrate suggests that the high concentrations of deposited NO we observe in the amnion of egg-type breeds are not essential, at least for supporting vital NO-dependent processes. As previously shown (Titov et al., 2018; Dolgorukova et al., 2020), the NO oxidation to nitrate occurs in the embryo tissues and mainly in muscle tissue. NO oxidation is practically absent in the liver and intestines. That is, we can assume that this oxidation is somehow associated with the development of muscle tissue. As for the role of NO deposited in the embryo of egg-type breeds, it may play a role as a pool in case of activation of NO oxidation processes, which can occur in any embryo.

Our analysis of the obtained data (Supplementary Table S2) shows that a high rate of NO oxidation is generally typical for meat-type and game chickens. It should be borne in mind that meat-type breeds are those that are profitable for raising birds for meat production, considering that they grow relatively quickly, and the gain in BW is ensured by relatively low feed costs. Note that the yield of gutted carcasses in broilers is only 5% higher than that in egg-type chickens. Therefore, the main feature for meat-type poultry is a rapid increase in BW. The breeds, lines and crosses listed in Supplementary Table S2 that had a high degree of NO oxidation in the embryos were also characterized by a more intensive growth of BW as compared to those with a lower degree of oxidation (Royter et al., 2005; Vinnikova and Titov, 2008). From our data (Supplementary Table S2), it also follows that NOD compounds are initially accumulated in the embryos. Starting from a certain point, these compounds begin to oxidize to nitrate. In egg-type embryos, NO oxidation is practically negligible. The key moments and processes of embryonic myogenesis are related to the fact that myotomes are laid down in E2 and E3 chick embryos, and the proliferation of myoblasts occurs up to E14. The process of NO oxidation in meat-type chick embryos occurs throughout the entire embryogenesis. Histological studies did not reveal any qualitative differences in the development of muscle tissues in BR vs. egg-type embryos characterized by respectively high and low rates of embryonic NO oxidation (Titov et al., 2018). It can be assumed that some factors associated with NO oxidation appear at E2 or E5 and this, apparently, is genetically determined and mediated by DGE of many genes involved in muscle development (Titov et al., 2018; Titov et al., 2020b).

**FIGURE 10:**
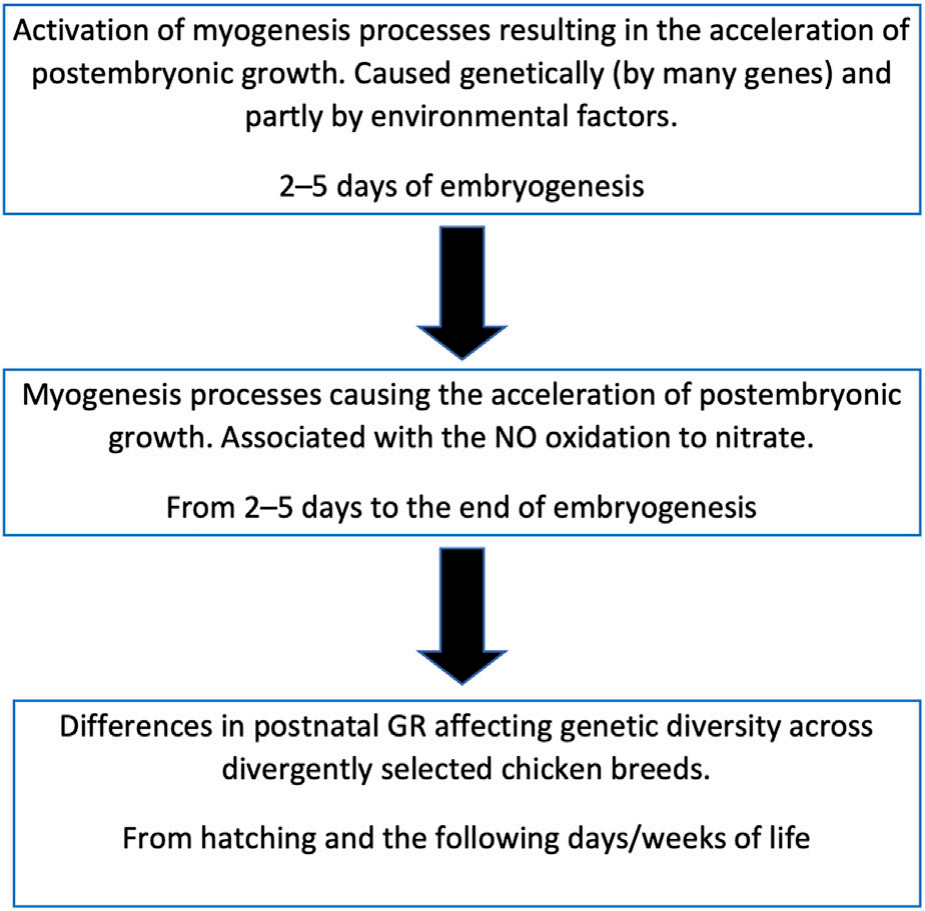
Scheme of relationship between the embryonic myogenesis processes, postnatal growth and genetic diversity in chicken breeds.

Cazzato et al. (2014) studied DGE of some important myogenesis associated genes at the earliest stages of embryogenesis and showed the effect of NOSI and NOD on DGE. According to our data, inhibition of NO synthase at the initial stage of embryogenesis by 80% did not significantly affect the postembryonic GR (Titov et al., 2018; Dolgorukova et al., 2020). Of interest is the difference not in the intensity of NO synthesis, which is approximately the same in all embryos of the same species, but in the degree of its oxidation that differs many times in fast-growing vs. slow-growing chickens. Therefore, our data suggest that: 1) NO oxidation degree is genetically determined and inherited; 2) it is determined by DGE of not one but many genes; and 3) there are chances for activation of NO oxidation in all avian embryos. It can also be assumed that it is not NO that primarily affects DGE, but DGE affects NO oxidation (Titov et al., 2020b). In other words, the process of NO oxidation can be triggered by internal genetic factors (Titov et al., 2018) and partially by external factors (Figure 10).

It can be hypothesized that NO oxidation is catalyzed by some heme-containing protein, similar to the process observed in the interaction of NO with oxyhemoglobin (Herold, 1999). What role NO oxidation itself plays is still not completely clear, however, this process can serve as a biochemical marker of the breed characteristics related to development (myogenesis) both in the embryonic and postembryonic periods (Dolgorukova et al., 2020). Being initiated at the beginning of embryo development, NO oxidation continues throughout the entire embryonic stages suggesting that, under the influence of genetically determined (and external) factors, a population of cells is formed, within which the oxidation process occurs. The exact mechanism of this intracellular interaction of different pathways may be associated with specific biochemical signaling networks (Bhalla and Iyengar, 1999) and should be studied further.

By confirming, and elucidating details of, some important phenomena in the chick embryo development, our findings expand the basic knowledge of how the early myogenesis genes work and how NO oxidation is involved in this process in various chicken breeds. Further investigation of these genetic signatures may have their practical significance as useful markers for the genetic breeding and genomic selection of chickens.

## 5 Conclusion

In the present study, we established that signatures of genetic diversity in divergently selected chicken breeds can be already traced at early developmental stages and be reflected in differences in embryonic myogenesis, NO metabolism, and postnatal growth patterns. Myogenesis associated genes were expressed in a coordinated manner, showing peculiar DGE and co-expression patterns depending on the type of muscle tissue under consideration (breast vs. thigh) and the type of divergent selection and utility to which this or that breed belonged. The coordinated (“accord”) expression patterns of the genes *MSTN*, *GHR*, and *MEFC2* in the breast and thigh muscles served as genetic diversity markers among the breeds under study. Additionally, related expression vectors for the *MYOG* and *MYOD1* genes in the breast muscles as well as *MYOG* and *MYF5* in the thigh muscles were discovered. It was demonstrated that the main part of NO synthesized in the avian embryo plays a specific role and can be accumulated in tissues as part of NOD compounds or be oxidized to nitrate. Being a biochemical marker for breed-specific characteristics that determine the rate of muscle mass growth (Titov et al., 2020b; Dolgorukova et al., 2020), NO oxidation correlated differently with early myogenesis in divergently selected breeds of different utility types: in BR embryos, NO was oxidized to nitrate by ∼90%, while in egg-type embryos, oxidation was minor. It is assumed that the degree of NO oxidation in embryonic tissues is genetically determined (Titov et al., 2018) and caused not by a specific gene but, apparently, by a combination of many DEG associated with the NO oxidation to nitrate. Postembryonic growth patterns correlated with features of early muscle development and NO metabolism were generally consistent with, and accurately captured, evolutionary history of divergently selected chicken breed types reflecting their existing genetic diversity.

## Data Availability

The original contributions presented in the study are included in the article/Supplementary Material, further inquiries can be directed to the corresponding author.
