# Supplementary material for: Unraveling signatures of chicken genetic diversity and divergent selection in breed-specific patterns of early myogenesis, nitric oxide metabolism and post-hatch growth
Source: Front Genet. 2023 Jan 11;13:1092242. doi: 10.3389/fgene.2022.1092242 (PMC9874007; doi:10.3389/fgene.2022.1092242)
Supplement: Supplementary file 1 [file DataSheet4.docx]

Supplementary Material

Supplementary Information SI3: Addition to the cluster analysis of the studied breeds based on differential gene expression data

# Type I (raw) dataset

The next step was to analyze the available matrices of raw expression data (i.e., primary FC values with preservation of their signs) in the breast (Table 2) and thigh (Table 3) muscles in the eight breeds using the ClustVis webtool (Metsalu and Vilo, 2015). Corresponding heat maps and breed clustering trees (Figure SI2-1B,D), as well as PCA plots using Euclidean distances (Figure SI3-1), were obtained. It can be seen that the patterns of PCA breed clustering in Figure SI3-1 for the Type I data in the breast and thigh muscles almost coincide with those in the scatter plots in Figure 1.

| **A** | **B** |
| --- | --- |

**Supplementary Figure SI3-1.** PCA plots for the distribution of the eight breeds according to the raw DGE for the seven genes in the breast **(A)** and thigh **(B)** muscles generated in the ClustVis program (Metsalu and Vilo, 2015). X and Y axes show principal component 1 (PC1) and principal component 2 (PC2) that explain 51.6% and 28.2% **(A)**, and 67.5% and 24.5% **(B)** of the total variance, respectively. N = 8 data points (breeds).

Clustering of breeds did not show clear patterns, with most breeds crowded and some of them scattered. The latter included YC, BB and UG on the PCA plot for the breast muscles (Figure SI3-1A), and YC and WC on the PCA plot for the thigh muscles (Figure SI3-1B). It is likely that the available raw expression data (Type I), which has not been mathematically processed and transformed, does not show a sufficient resolution “sensitivity” for detecting breed clustering patterns by two different methods (i.e., PCA and clustering on heatmaps). Apparently, this is due to the presence of a large scatter (by sign, amplitude, and order of differences) in the obtained FC values. Therefore, the question arose about the selection of an adequate approach to the transformation of raw data, which would allow us to obtain more plausible clustering patterns, as described in the section **2 Materials and methods** (see subsection **2.2 DGE assessment**).

In the above rough analysis of raw data using the PCA method, of particular concern was a “crowding” of the four breeds BR, OMF, LR and WC for the breast muscle data (Figure SI3-1A), and of the five breeds BB, BR, PRW, OMF and LR for the thigh muscle data (Figure SI3-1B ), i.e., those that were created, as a rule, through the multidirectional (divergent) selection and represent different evolutionary branches of domestic chickens (Moiseyeva et al., 2003; Larkina et al., 2021). This “crowding” pattern was also reflected in the corresponding heatmaps (Figures SI2-1B and SI2-1D).

# Transformed Type IIb dataset

Of particular interest was an attempt to combine the Type IIb data for the breast and thigh muscles into one matrix and analyze them using the Phantasus web server program. While PCA (Figure SI3-2) did not demonstrate a meaningful discriminatory power to distinguish between the two (breast and thigh) muscle tissue types or between the breeds studied, hierarchical clustering (Figure SI3-3) showed a more plausible pattern of interstitial and interbreed differences. Thereby, data for one type of tissue were clustered, as a rule, together. The exception was representatives of the egg (LR) and game (UG) types, in which the gene expression data for the breast or femoral thigh constituted common independent (separately standing) clusters. This could indicate that early myogenesis in these breeds proceeds somewhat differently than in other breeds of meat and dual purpose types.


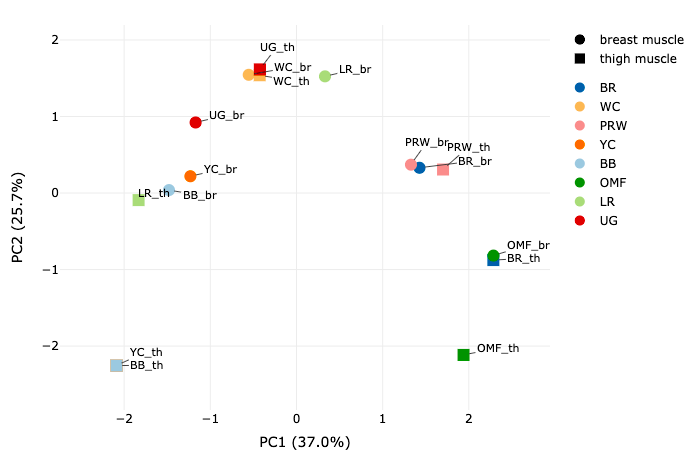


**Supplementary Figure SI3-2.** PCA plot using the Type IIb data as inferred for the eight studied breeds and seven tested myogenesis associated genes expressed in the breast (_br) and thigh (_th) muscles as generated in the Phantasus program (Zenkova et al., 2018). X and Y axes show principal component 1 (PC1) and principal component 2 (PC2) that explain 37.0% and 25.7% of the total variance, respectively. N = 8 data points (breeds).


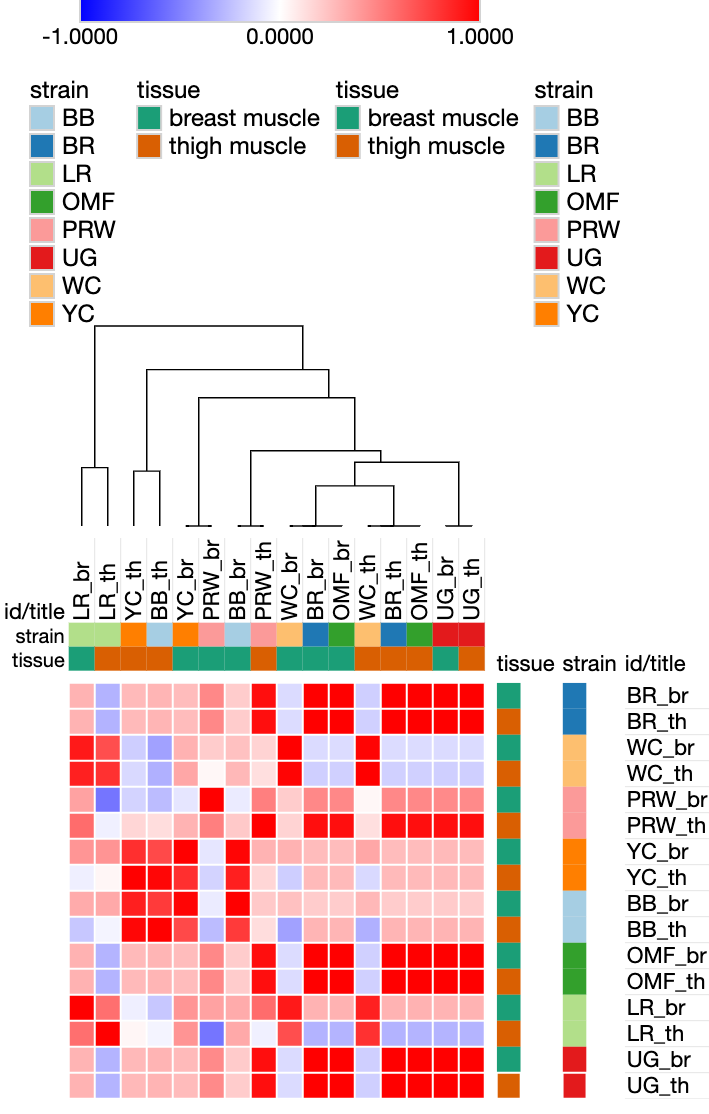


**Supplementary Figure SI3-3.** Heatmap and hierarchical clustering tree derived in Phantasus (Zenkova et al., 2018) from the Pearson correlation-based similarity matrix and using the Type IIb data as inferred for the eight studied breeds and seven tested myogenesis associated genes expressed in the breast (_br) and thigh (_th) muscles. For clustering, matrix values (for a precomputed similarity matrix) were applied as metrics (with the average linkage method option selected).

# Transformed Type IIIa and IIIb datasets

The matrices for the two Type IIIa and IIIb datasets were alternately employed to build phylogenetic trees using the Neighbor Joining method (Figures SI3-4A,C and SI3-4B,D, respectively).

| 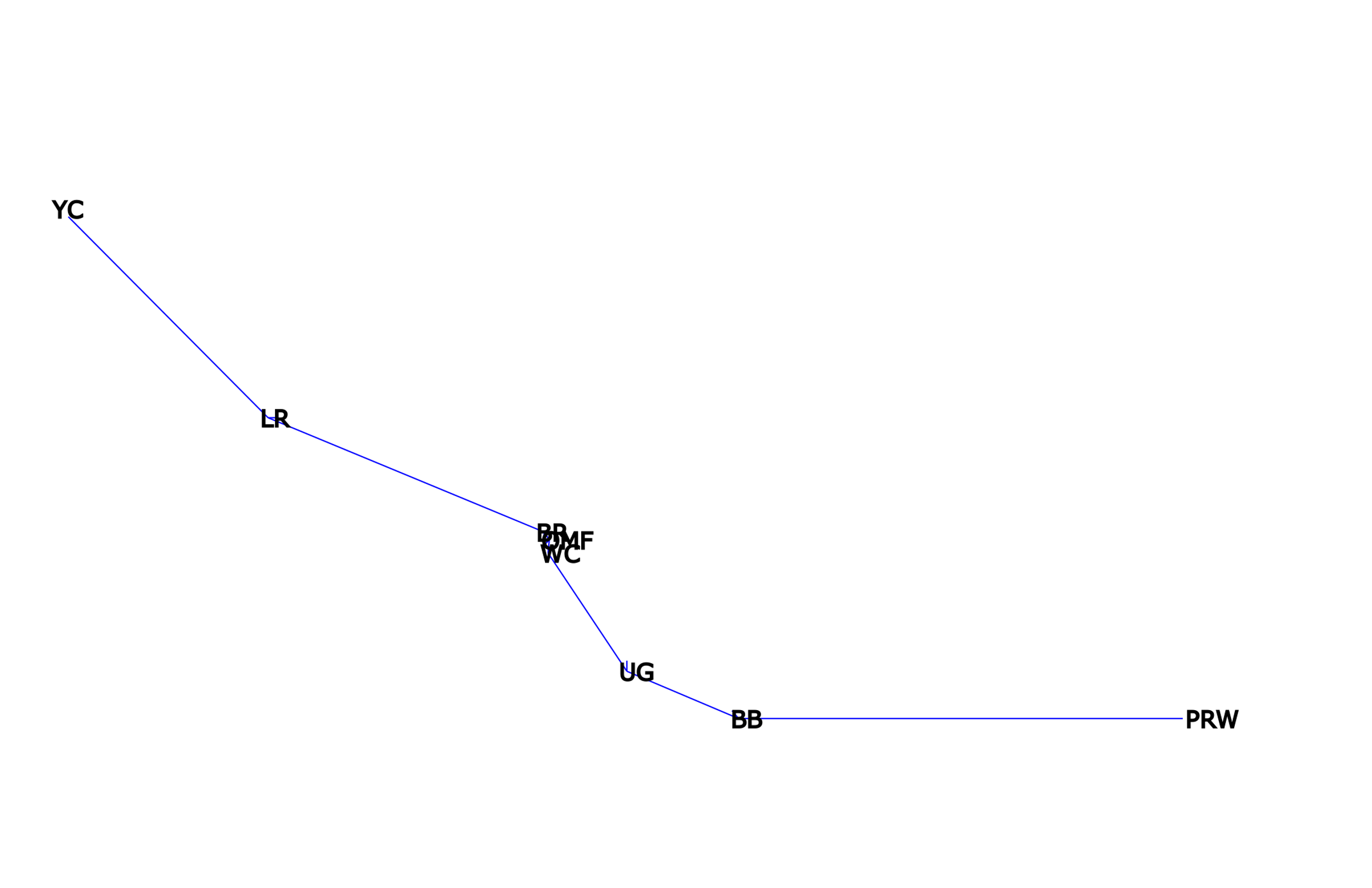 | 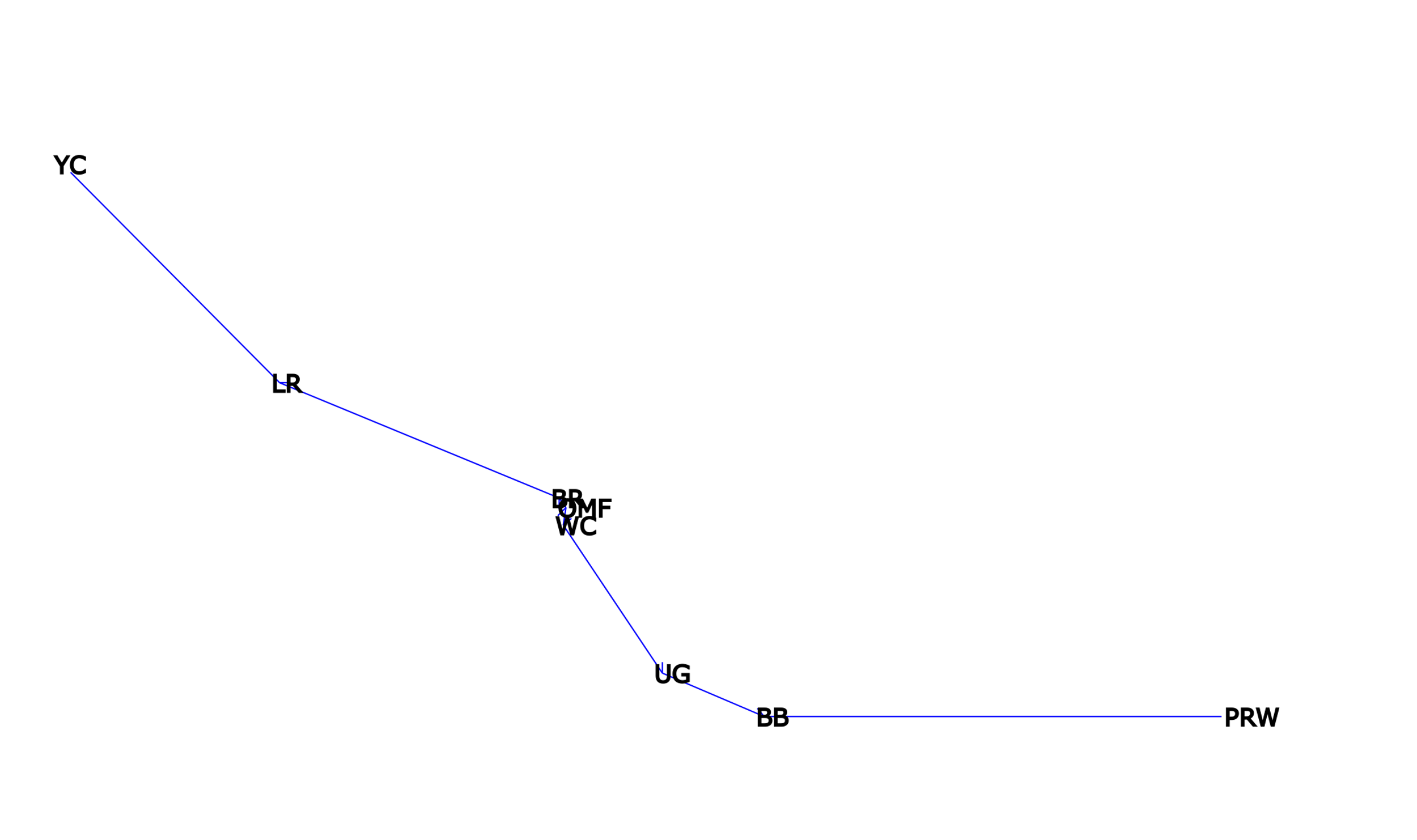 |
| --- | --- |
| **A** | **B** |
| 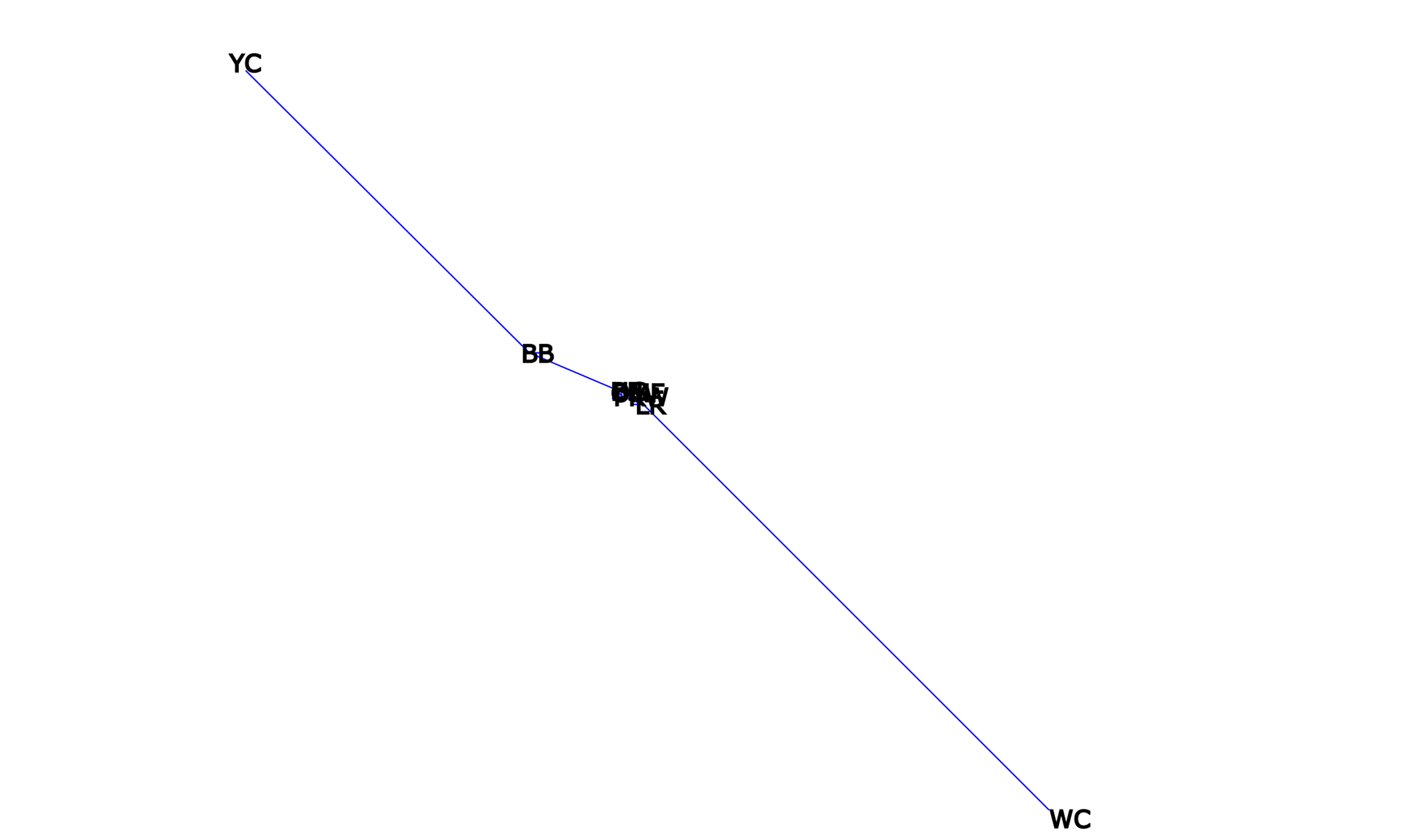 | 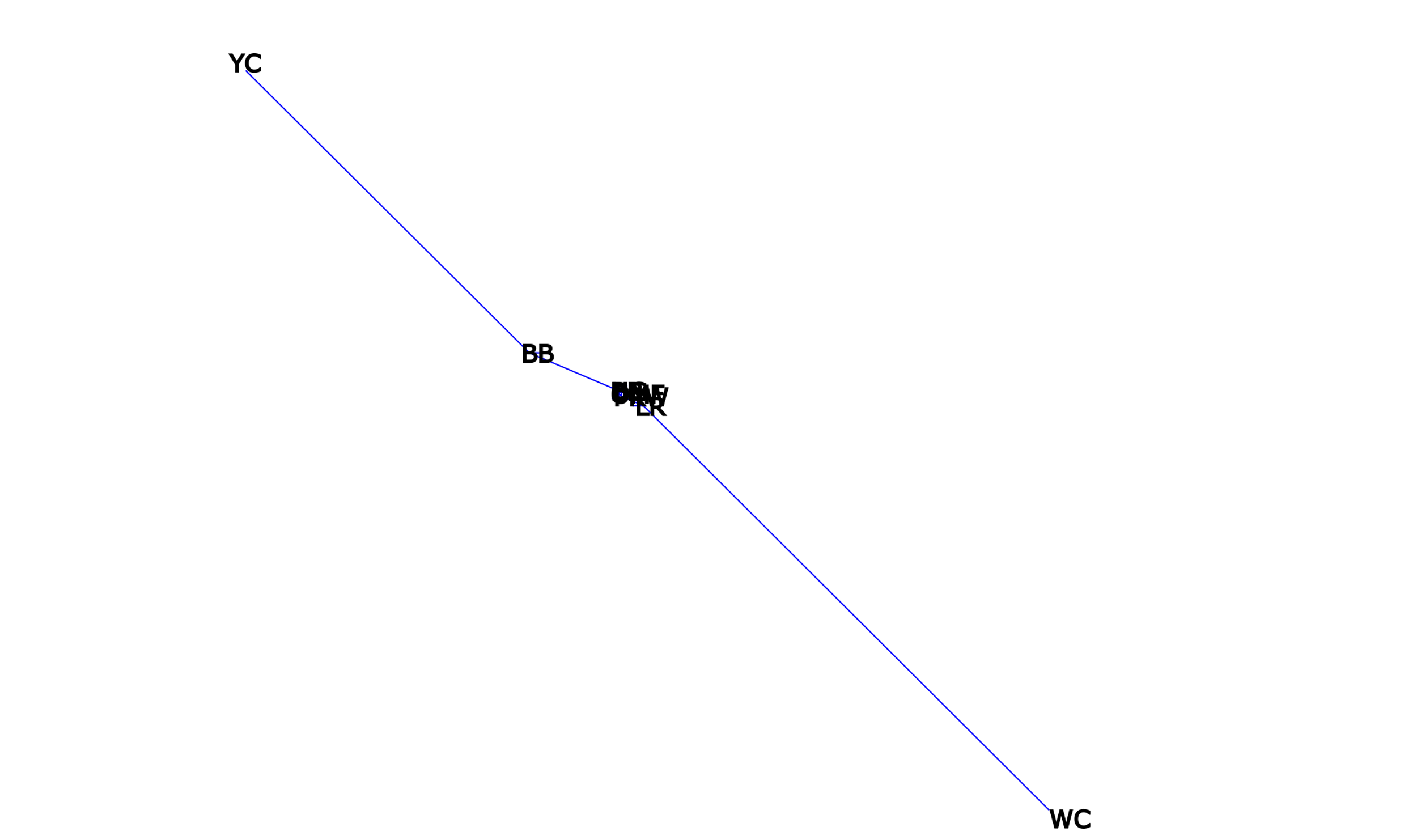 |
| **C** | **D** |

**Supplementary Figure SI3-4.** Radial Neighbor Joining trees with proportional edge lengths using the Type IIIa data (A, C) and IIIb (B, D) as inferred for the eight studied breeds and seven tested myogenesis associated genes expressed in the breast (A, B) and thigh (C, D) muscles using the T-REX tool (Boc et al., 2012).

As you can see, the radial topology of trees with proportional lengths of edges turned out to be almost identical when based on the Type IIIa (Figure SI3-4A,C) and Type IIIb (Figure SI3-4B,D) datasets. At the same time, we note that the formulas of the corresponding trees (in the Newick format; see Figure SI3-5) had some numerical differences, but the proportionality of the trees themselves was practically the same. Figure SI3-5 also shows radial topology trees without proportional lengths of edges.

| 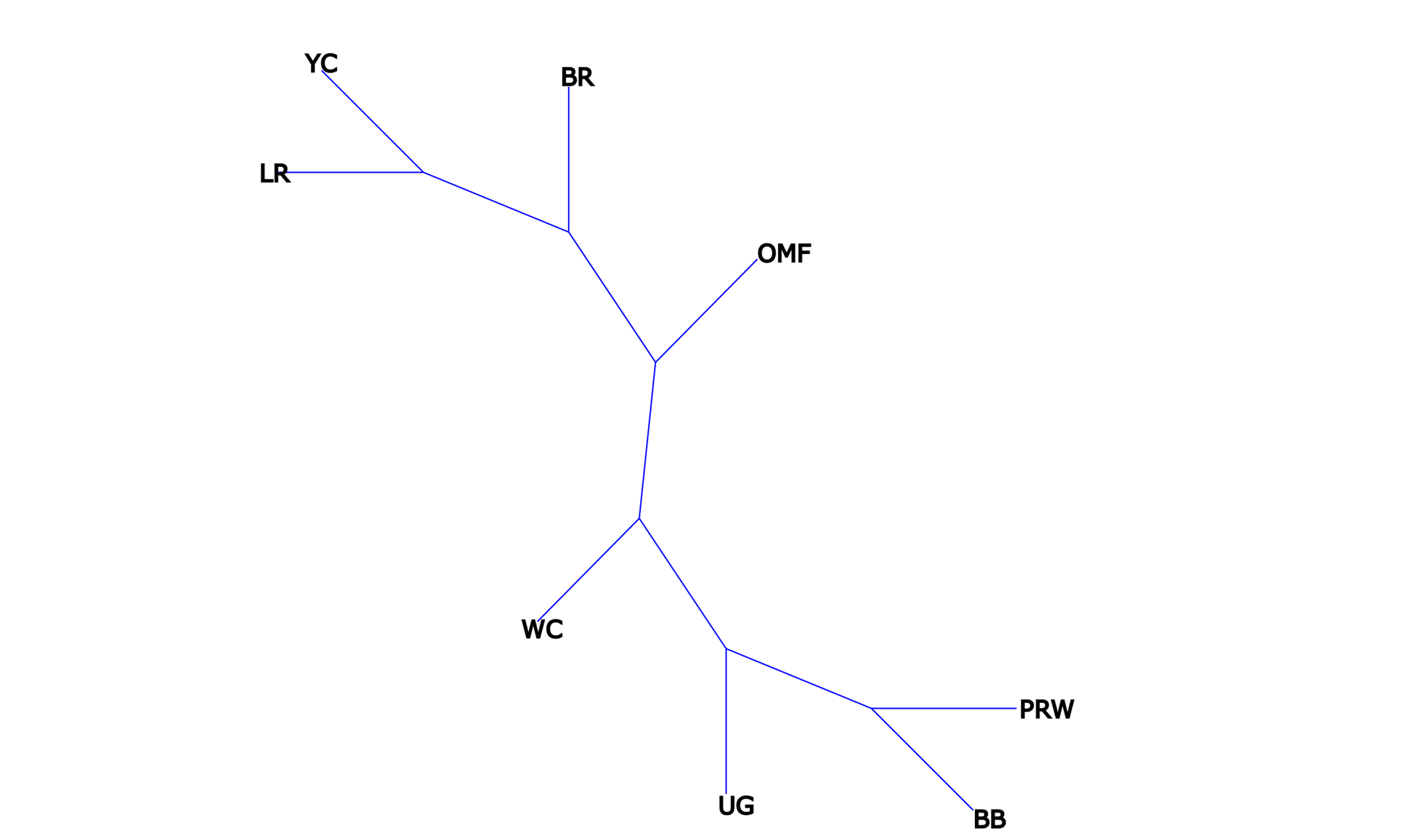 | | 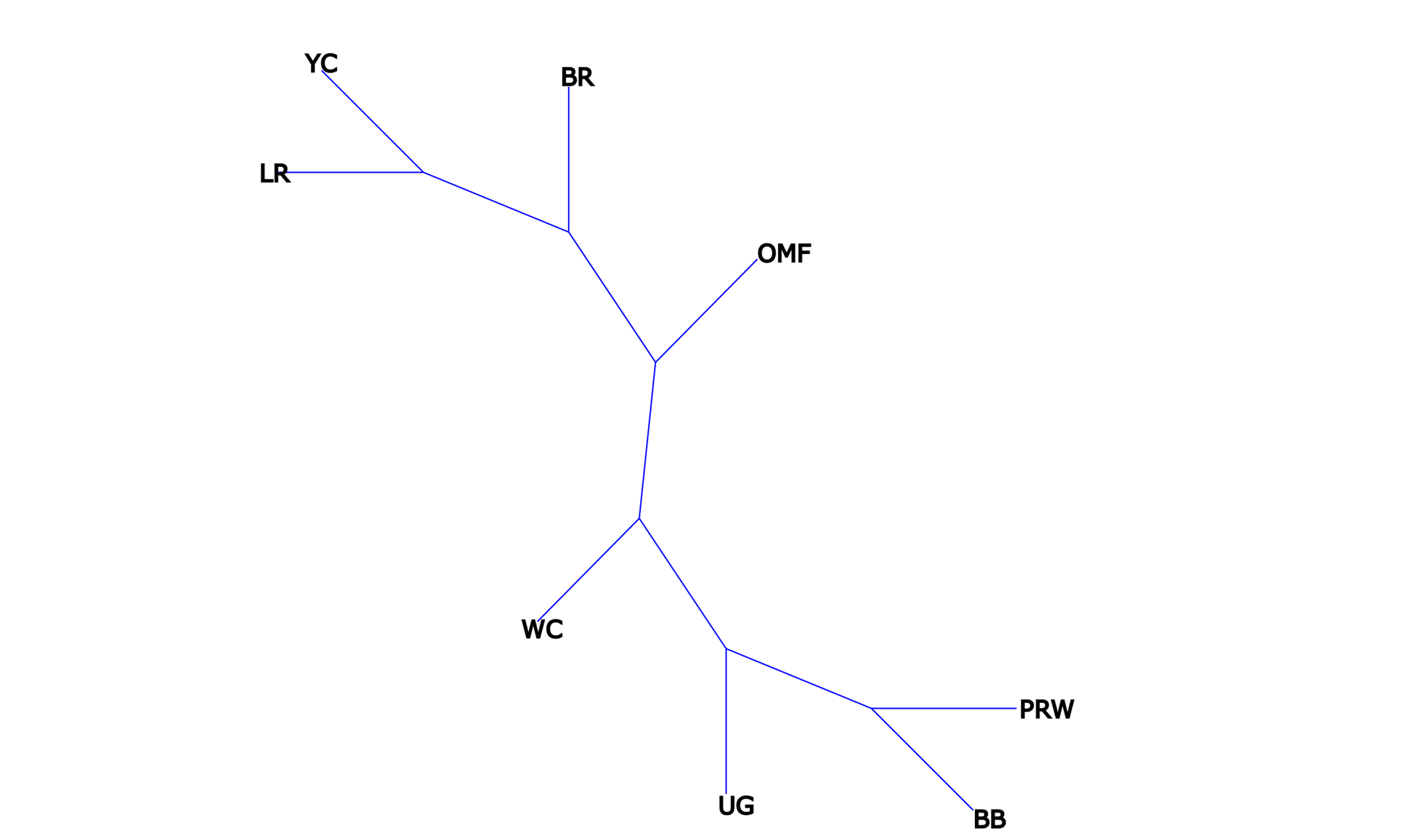 |
| --- | --- | --- |
| **A** | | **B** |
| 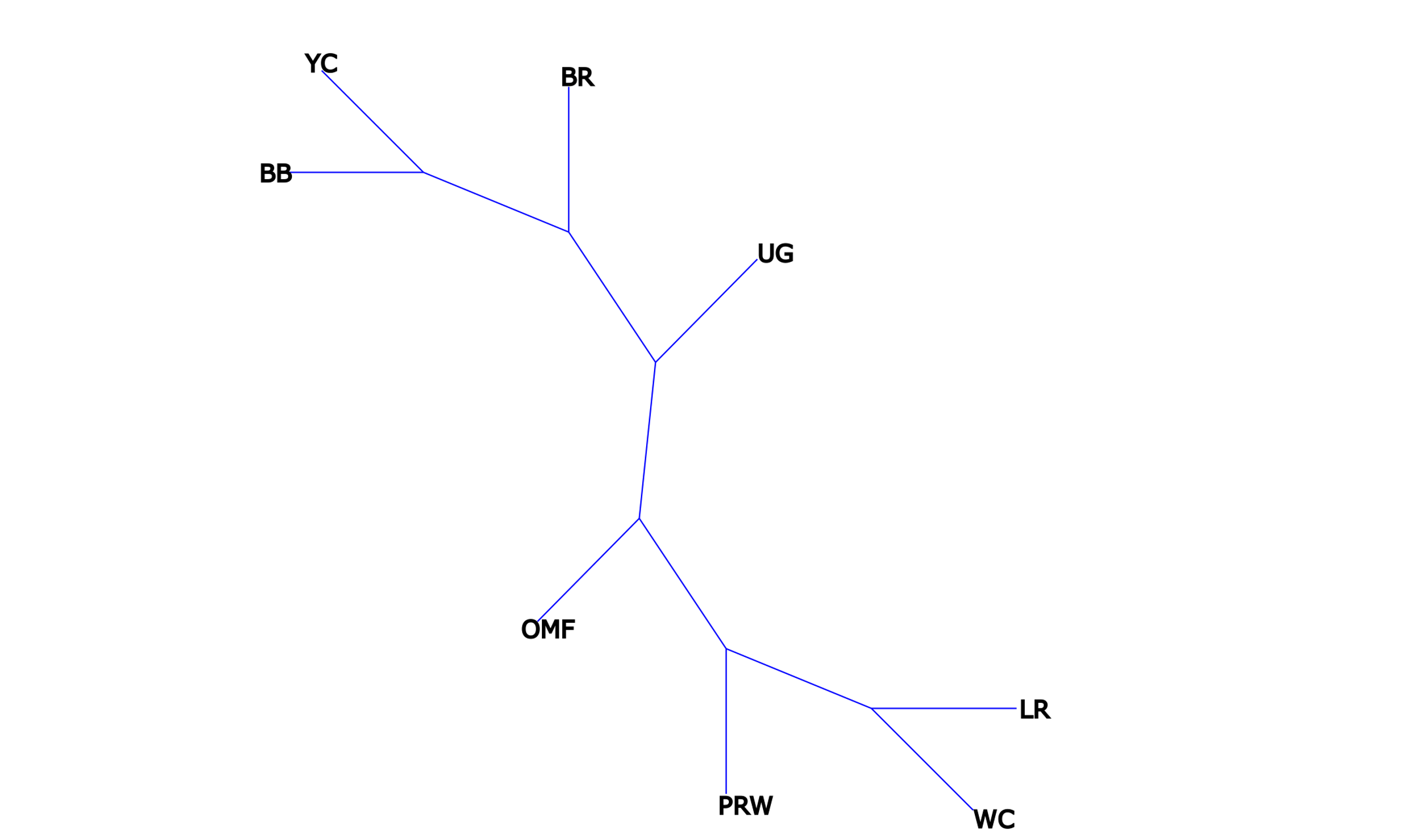 | | 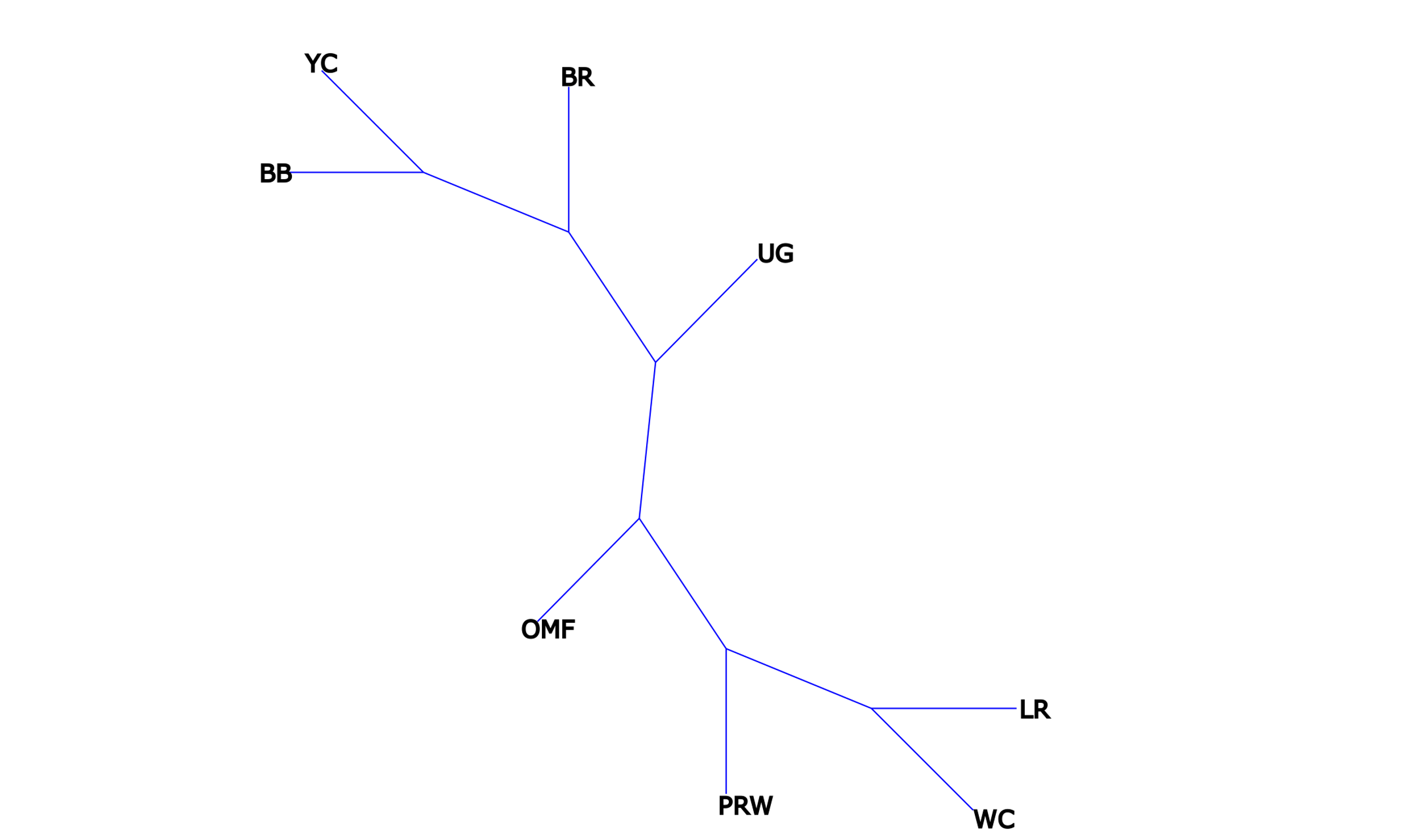 |
| **C** | **D** | |

**Supplementary Figure SI3-5.** Radial trees without proportional lengths of edges using the Type IIIa **(A, C)** and IIIb data **(B, D)** as inferred for the eight studied breeds and seven tested myogenesis associated genes expressed in the breast **(A, B)** and thigh **(C, D)** muscles using the T-REX tool (Boc et al., 2012).

Trees in the Newick format:
**(A)** (WC:0.0000,(OMF:0.0000,(BR:0.0000,(YC:4375.0506,LR:0.0000):4427.2793):149.3542):206.0300,(UG:0.0000,(BB:0.0000,PRW:6747.8798):1802.4177):2113.3247);
**(B)** (WC:0.0000,(OMF:0.0000,(BR:0.0000,(YC:633.1582,LR:0.0000):622.2784):24.0893):37.5705,(UG:0.0000,(BB:0.0000,PRW:962.6699):227.1518):366.5311);
**(C)** (OMF:0.0000,(UG:0.0000,(BR:0.0000,(YC:0.0509,BB:0.0000):0.0121):0.0000):0.0002,(PRW:0.0000,(WC:0.0723,LR:0.0000):0.0030):0.0005);
**(D)** (OMF:0.0000,(UG:0.0000,(BR:0.0000,(YC:0.0729,BB:0.0000):0.0173):0.0002):0.0003,(PRW:0.0000,(WC:0.1036,LR:0.0000):0.0043):0.0006);

It seems that it is the radial topology with proportional lengths of edges (Figure SI3-4) that can better reflect the real breed relationship pattern. At the same time, in the case of the breast muscles (Figure SI3-4A,B), it was possible to remove the LR from the crowded “core” (BR, OMF, WC), which is very well consistent with the breed characteristics of the egg-type chickens, since it shows that it still has its own specific pattern of embryonic expression of myogenesis associated genes. This indicates a better resolution of the converted data analysis as compared to when the previous raw data analysis (Figure SI3-1) found that the egg-type birds were seemingly “identical” to the broilers. The fact that BR and WC are included in the crowded “core” in the center of the plot (Figure SI3-4A,B) also very well reflects the breed characteristics of the meat-type poultry, since the WC meat-type breed is the direct paternal line of the BR broiler cross, that is, their early myogenesis is expected to coincide. As for the entry of OMF into the “core” of crowding, it can be assumed that somehow this breed turned out to be so close to the broiler chickens in terms of the pattern of embryonic expression of myogenesis associated genes, or the Neighbor Joining method still had insufficient resolving power and could not adequately reflect interbreed differences for this dataset.

Let us also point out the specificity of the expression of myogenesis associated genes in the breast muscles in PRW that is the maternal line of the BR broiler cross, which was expressed in the isolation and remoteness of this breed on the corresponding phylogenetic tree (Figure SI3-4A,B). At the same time, in the case of the thigh muscles (Figure SI3-4C,D), a very specific pattern of early expression of myogenesis associated genes was observed in the WC meat-type breed, the paternal line of the BR broiler cross. Apparently, such a contrast ratio of the action of myogenesis associated genes in the breast and thigh muscles in the parental lines favorably affects myogenesis and the rate of muscle growth in BR chickens.

Interestingly, two breeds, BB and especially YC, also showed isolation and remoteness from other breeds in the expression of myogenesis associated genes in both breast (Figure SI3-4A,B) and thigh muscles (Figure SI3-4C,D).
