# Supplementary material for: Unraveling signatures of chicken genetic diversity and divergent selection in breed-specific patterns of early myogenesis, nitric oxide metabolism and post-hatch growth
Source: Front Genet. 2023 Jan 11;13:1092242. doi: 10.3389/fgene.2022.1092242 (PMC9874007; doi:10.3389/fgene.2022.1092242)
Supplement: Supplementary file 2 [file DataSheet3.docx]

Supplementary Material

Supplementary Figures


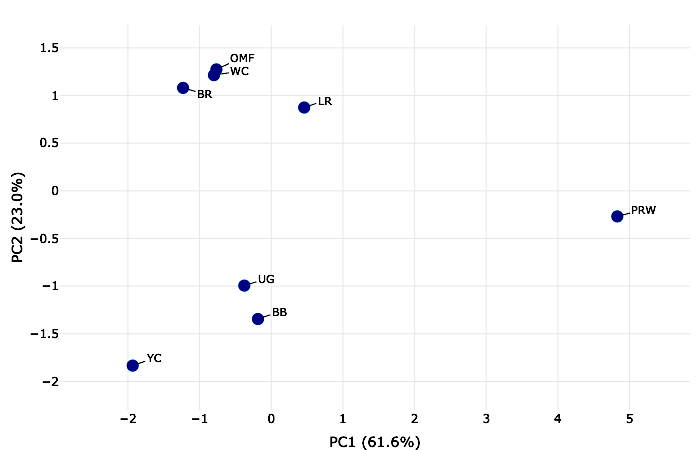

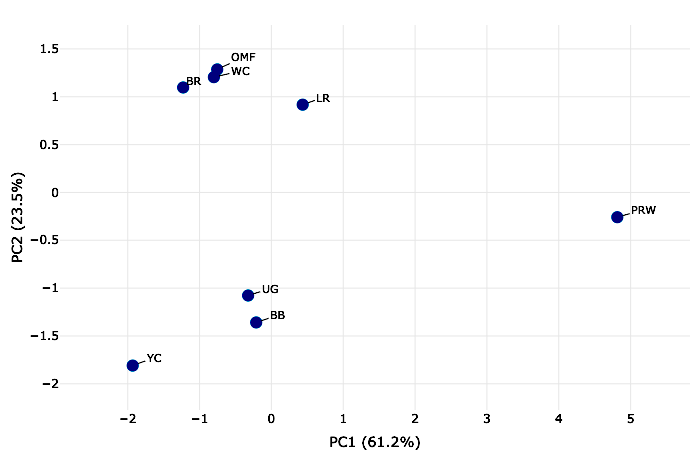


**A** **B**


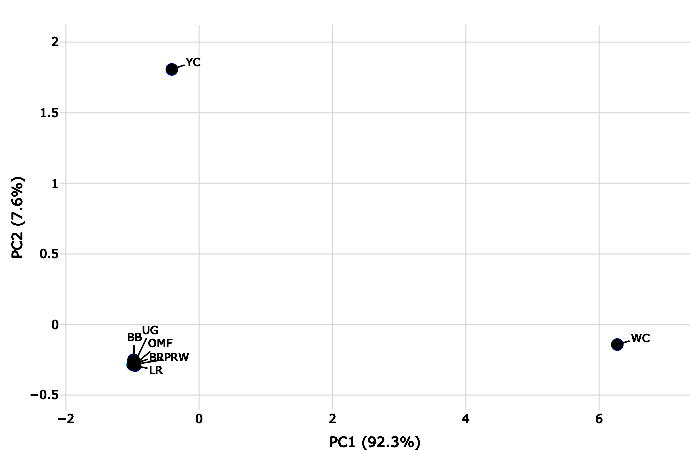

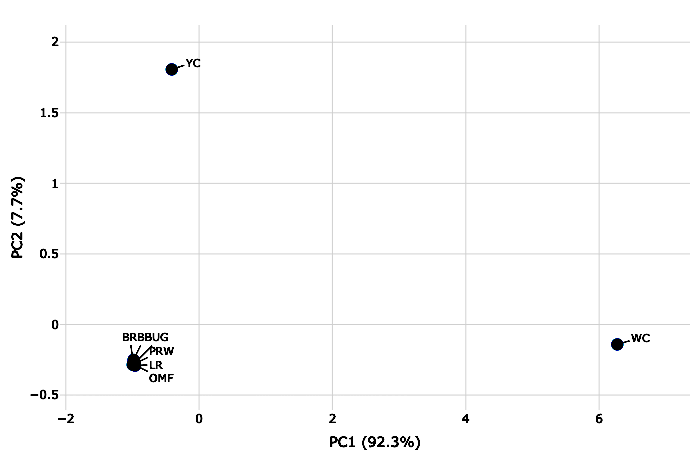


**C** **D**

**Supplementary Figure S1.** PCA plots generated using the Phantasus tool (Zenkova et al., 2018) and the Type IIIa **(A, C)** and IIIb **(B, D)** data as inferred for the eight studied breeds and seven tested myogenesis associated genes expressed in the breast **(A, B)** and thigh **(C, D)** muscles. For PCA analysis, similarity matrices were used based on the Euclidean metric. X and Y axes show principal component 1 (PC1) and principal component 2 (PC2) that explain respective percentage values of the total variance. N = 8 data points (breeds).

**Supplementary Figure S2.** Heatmap and clustering trees for 13 breeds based on early growth traits, including egg weight and body weight of chicks at three ages, as generated in the ClustVis software (Metsalu and Vilo, 2015) using Euclidean distances (with the average option selected as linkage method).


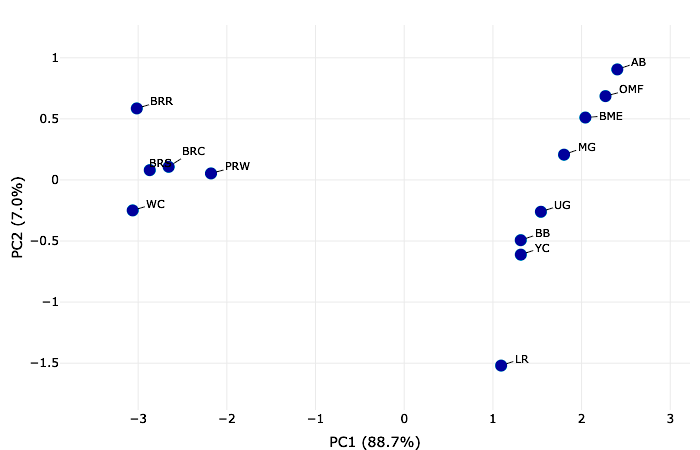

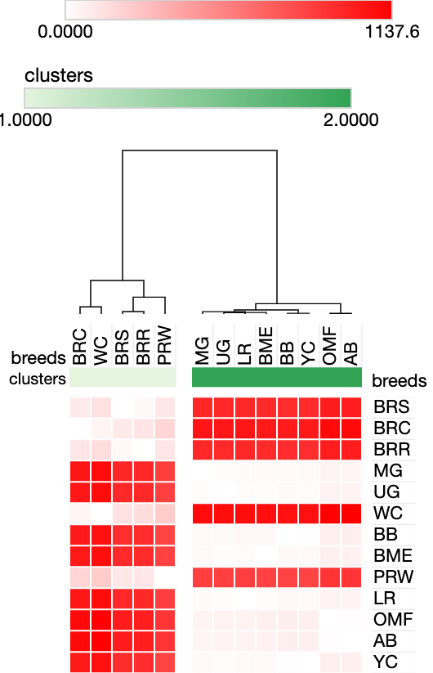


**A** **B**

**Supplementary Figure S3.** Analysis of the distribution of 13 breeds by postnatal growth traits, including egg weight, body weight of chicks at three ages, and growth rate over two periods, as performed using the Phantasus software (Zenkova et al., 2018). **(A)** PCA plot. X and Y axes show principal component 1 (PC1) and principal component 2 (PC2) that explain 88.7% and 7.0% of the total variance, respectively. N = 13 data points (breeds). **(B)** Heatmap and hierarchical clustering tree using Euclidean distance-based similarity matrix. For clustering, matrix values (for a precomputed distance matrix) were applied as metrics (with the average linkage method option selected).


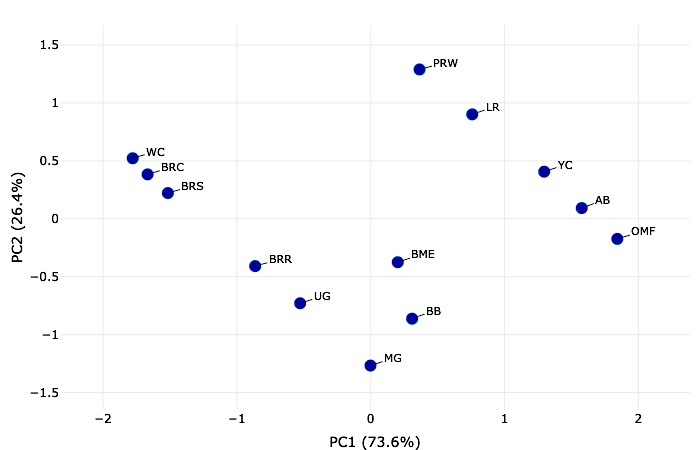

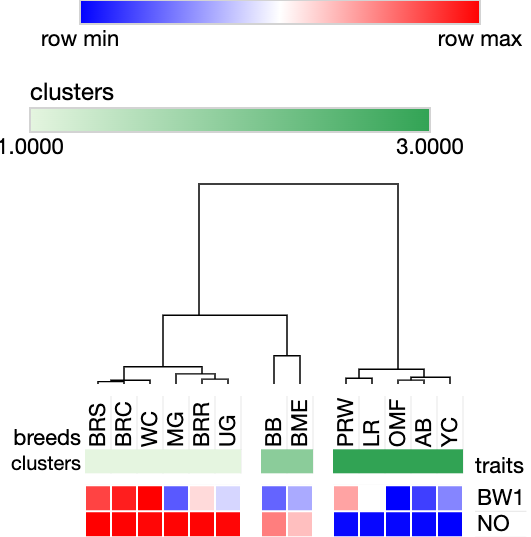


**A** **B**

**Supplementary Figure S4.** Analysis of the distribution of 13 breeds according to the characteristics of E7 oxidation of NO and body weight at 1 day old (BW1) as performed in the Phantasus program (Zenkova et al., 2018). **(A)** PCA plot. X and Y axes show principal component 1 (PC1) and principal component 2 (PC2) that explain 73.6% and 26.4% of the total variance, respectively. N = 13 data points (breeds). **(B)** Heatmap and hierarchical clustering tree based on Euclidean distance metric (with the average linkage method option selected).


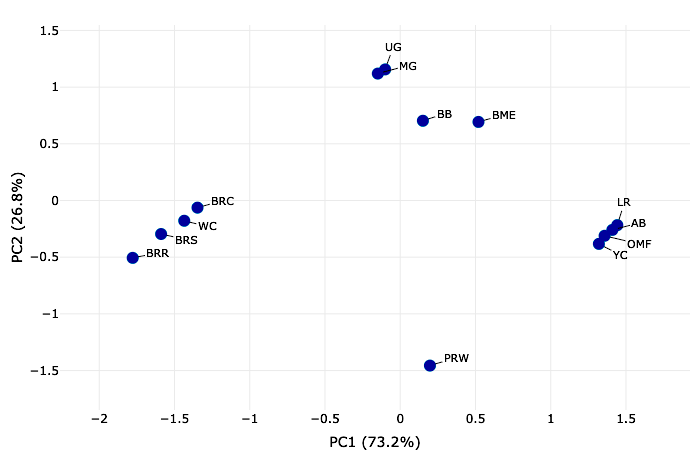

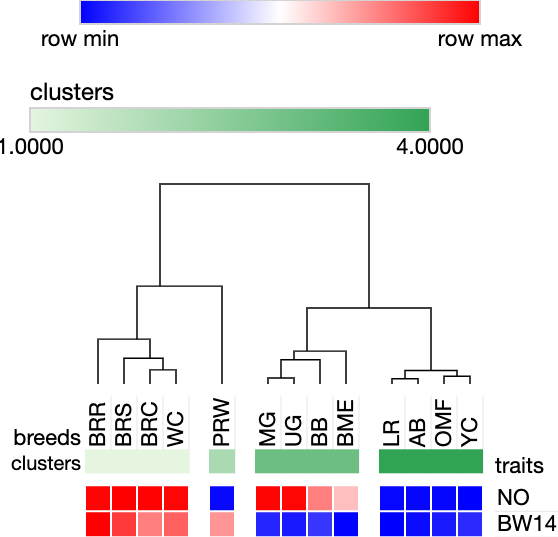


**A** **B**

**Supplementary Figure S5.** Analysis of the distribution of 13 breeds according to the characteristics of E7 oxidation of NO and body weight at 14 days of age as performed in the Phantasus program (Zenkova et al., 2018). **(A)** PCA plot. X and Y axes show principal component 1 (PC1) and principal component 2 (PC2) that explain 73.2% and 26.8% of the total variance, respectively. N = 13 data points (breeds). **(B)** Heatmap and hierarchical clustering tree based on Euclidean distance metric (with the average linkage method option selected).

**Supplementary Figure S6.** Heatmap and clustering trees for 13 breeds based on E7oxidation of NO and body weight at 14 days of age as performed in the ClustVis program (Metsalu and Vilo, 2015) using Euclidean distances (with the average option selected as linkage method).

**Supplementary Figure S7.** Heatmap and clustering trees for 13 breeds based on E7 oxidation of NO and postnatal growth, including egg weight and body weight of chicks at three ages, as performed in the ClustVis program (Metsalu and Vilo, 2015) using Euclidean distances (with the average option selected as linkage method).

**Supplementary Figure S8.** Spearman correlation values in pairwise comparison of E7 oxidation of NO (NO) and postnatal growth, including egg weight (EW) and body weight of chickens at 1-, 14-, and 28-day-old (BW1, BW14 and BW28) in 13 breeds. Significant correlations are not crossed out with “×” (*p* < 0.05).


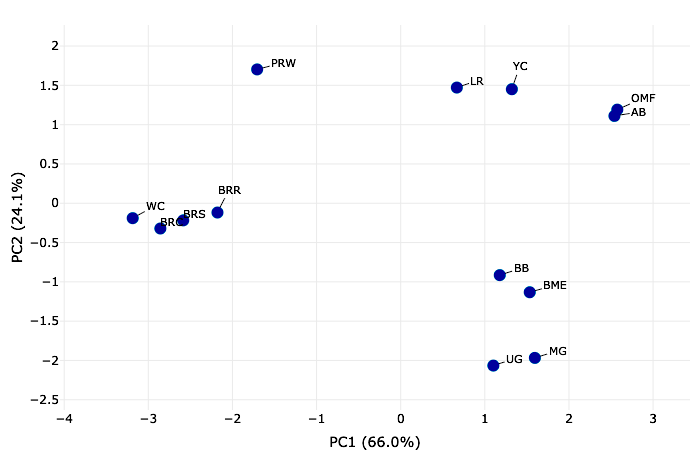

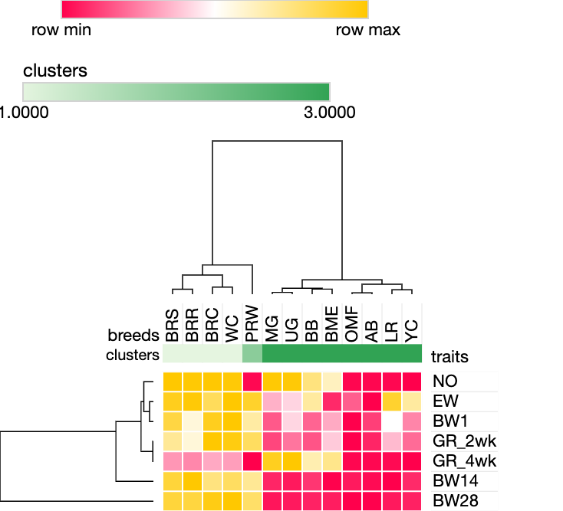


**A** **B**

**Supplementary Figure S9.** Analysis of the distribution of 13 breeds by traits of E7 oxidation of NO and postnatal growth, including egg weight, body weight of chicks at three ages, and growth rate over two periods, as performed in the Phantasus software (Zenkova et al., 2018). **(A)** PCA plot. X and Y axes show principal component 1 (PC1) and principal component 2 (PC2) that explain 66.0% and 24.1% of the total variance, respectively. N = 13 data points (breeds). **(B)** Heatmap and hierarchical clustering tree based on Euclidean distance metric (with the average linkage method option selected).

**Supplementary Figure S10.** Heatmap and clustering trees of 13 breeds for E7 oxidation of NO and postnatal growth, including egg weight, body weight of chicks at three ages and growth rate for two periods, as performed in the ClustVis program (Metsalu and Vilo, 2015) using Euclidean distances (with the average option selected as linkage method).

**Supplementary Figure S11.** The PCA plot resulted from an analysis of the relationships of the seven traits (E7 oxidation of NO and postnatal growth, including egg weight, body weight of chicks at three ages, and growth rate over two periods) among 13 breeds as performed in the ClustVis program (Metsalu and Vilo, 2015). Unit variance scaling is applied to rows; singular value decomposition with imputation is used to calculate principal components. X and Y axes show principal component 1 (PC1) and principal component 2 (PC2) that explain 95.6% and 2.7% of the total variance, respectively. N = 7 data points (traits).

**A** **B**

**Supplementary Figure S12.** Spearman correlation values in pairwise comparison of DGE in the breast **(A)** and thigh **(B)** muscles and postnatal growth, including egg weight (EW) and body weight of chickens at 1-, 14- and 28-day-old (BW1, BW14 and BW28) in the eight breeds. Significant correlations are not crossed out with “×” (*p* < 0.05).

**A** **B**

**Supplementary Figure S13.** Distribution of the eight breeds based on the analysis of relationships of 19 traits (DGE of myogenesis associated genes and NO metabolism in embryos, as well as indicators of early growth of chickens) performed in the ClustVis program (Metsalu and Vilo, 2015). **(A)** PCA plot. Unit variance scaling is applied to rows; singular value decomposition with imputation is used to calculate principal components. X and Y axes show principal component 1 (PC1) and principal component 2 (PC2) that explain 46.2% and 23.7% of the total variance, respectively. N = 8 data points (breeds). **(B)** Heatmap and clustering trees using Euclidean distances (with the average option selected as linkage method).


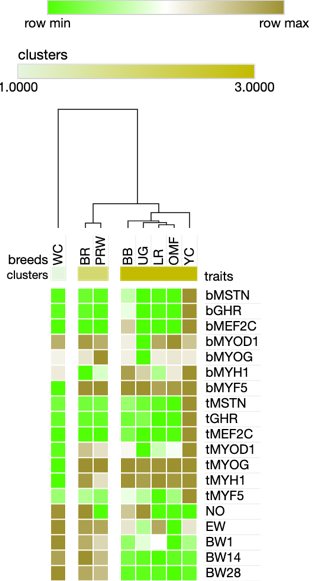


**A**


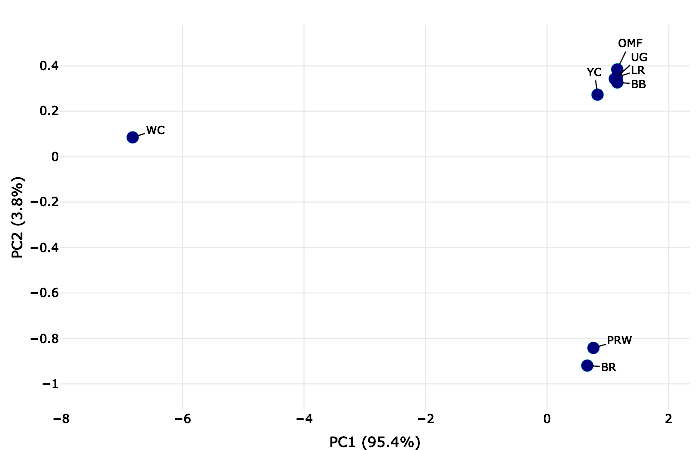

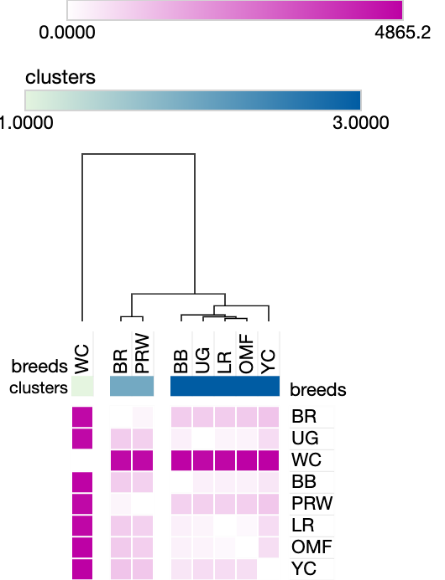


**B** **C**

**Supplementary Figure S14.** Distribution of eight breeds based on the analysis of relationships of 19 traits (DGE of myogenesis associated genes and NO metabolism in embryos, as well as indicators of early growth of chicks) performed in the Phantasus program (Zenkova et al., 2018). (A) Heatmap and hierarchical clustering tree based on Euclidean distance metric (with average linkage method). (B) PCA plot based on Euclidean distance-based similarity matrix. X and Y axes show principal component 1 (PC1) and principal component 2 (PC2) that explain 95.4% and 3.8% of the total variance, respectively. N = 8 data points (breeds). (C) Heatmap and hierarchical clustering tree using Euclidean distance metric (with average linkage method) and based on precomputed Euclidean distance-based similarity matrix.


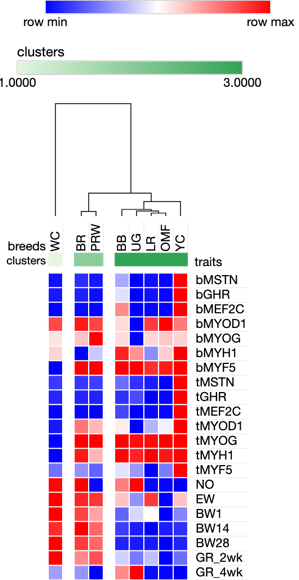


**A**


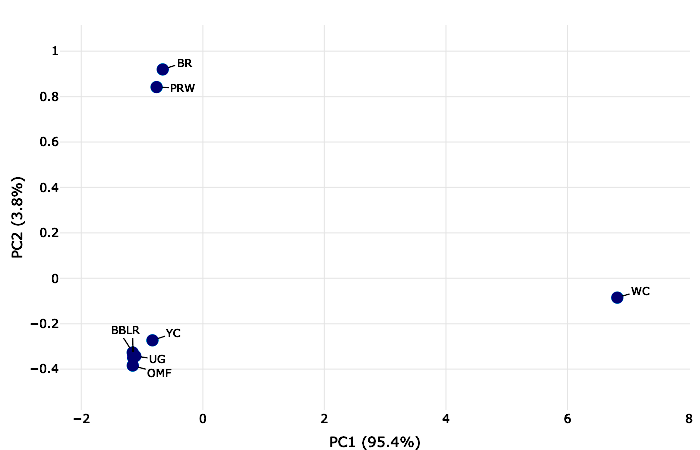

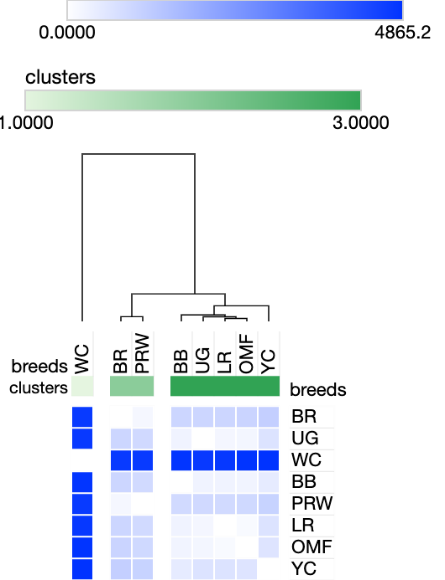


**B** **C**

**Supplementary Figure S15.** Distribution of the eight breeds based on the analysis of the relationships of 21 traits (DGE of myogenesis associated genes and NO metabolism in embryos, as well as indicators of early growth of chickens) as performed in the Phantasus program (Zenkova et al., 2018). **(A)** Heatmap and hierarchical clustering tree based on Euclidean distance metric (with average linkage method). **(B)** PCA plot based on Euclidean distance-based similarity matrix. X and Y axes show principal component 1 (PC1) and principal component 2 (PC2) that explain 95.4% and 3.8% of the total variance, respectively. N = 8 data points (breeds). **(C)** Heatmap and hierarchical clustering tree using Euclidean distance metric (with average linkage method) and based on precomputed Euclidean distance-based similarity matrix.

**Supplementary Figure S16.** PCA plot resulted from the analysis of relationships of 21 traits (differential expression of myogenesis associated genes and NO metabolism in embryos, as well as indicators of early growth of chickens) in the eight breeds, performed using the ClustVis program (Metsalu and Vilo, 2015). Unit variance scaling is applied to rows; singular value decomposition with imputation is used to calculate principal components. X and Y axes show principal component 1 (PC1) and principal component 2 (PC2) that explain 66.3% and 14.3% of the total variance, respectively. N = 21 data points (traits).

**A** **B**

**Supplementary Figure S17.** Spearman correlation values in pairwise comparison of **(A)** the eight breeds (taking into account 21 traits) and **(B)** 21 indicators (in eight breeds), including differential gene expression in chest (b…) and thigh (t…) muscles, E7 oxidation of NO (NO), egg weight (EW), body weight of chicks at 1, 14 and 28 days of age (BW1, BW14 and BW28) and growth rate in the first two (GR2wk) and four (GR4wk) weeks of life. Significant correlations are not crossed out with “×” (*p* < 0.05).
