# Supplementary material for: Unraveling signatures of chicken genetic diversity and divergent selection in breed-specific patterns of early myogenesis, nitric oxide metabolism and post-hatch growth
Source: Front Genet. 2023 Jan 11;13:1092242. doi: 10.3389/fgene.2022.1092242 (PMC9874007; doi:10.3389/fgene.2022.1092242)
Supplement: Supplementary file 3 [file DataSheet5.docx]

Supplementary Material

Supplementary Tables

**Table S1.** Primer pairs used for quantitative real-time PCR analysis.

| **Name** | **Symbol** | **Primer sequence** |
| --- | --- | --- |
| Myostatin | *MSTN* | F: 5′-TTTAGAGGTCAGAGTTACAGACAC-3′  R: 5′-TTTAGGTGCTATAATCCAGTCCCA-3′ |
| Growth hormone receptor | *GHR* | F: 5′-CAGATACTGACAGGCTCCTGAGT-3′  R: 5′-GAGATGGCATCACATGTGTCGCT-3′ |
| Myocyte-specific enhancer factor 2c | *MEF2C* | F: 5′-AGCAGCTCAGCCACTTTCTC-3′  R: 5′-AATATTCACCACCCGGTTCA-3′ |
| Myogenic differentiation 1 | *MYOD1* | F: 5′-CGTGAGCAGGAGGATGCATA-3′  R: 5′-GGGACATGTGGAGTTGTCTG-3′ |
| Myogenin | *MYOG* | F: 5′-AGCCTCAACCAGCAGGAG-3′  R: 5′-TGCGCCAGCTCAGTTTTGGA-3′ |
| Myosin | *MYH1* | F: 5′-AGACAAAACACCTGGTGCC-3′  R: 5′-CCTCCTCTCCACCATTCTTG-3′ |
| Myogenic factor 5 | *MYF5* | F: 5′-TGCCCTGAGGAAGAGGAACAC-3′  R: 5′-ACGATGCTGGAGAGGCAGTC-3′ |
| TATA-binding protein | *TBP* | F: 5′-AGCTCTGGGATAGTGCCACAG  R: 5′-ATAATAACAGCAGCAAAACGCTTG-3′ |

F: forward; R: reverse.

**Table S2.** Means (M ± m) of egg weight, body weight of chickens at three ages (1, 14 and 28 days) and the degree of NO oxidation to nitrate in homogenates of 7-day-old embryos in 13 chicken breeds.

| **Breed** | **Code** | **Type^1^** | **Egg weight, g** | **Body weight of chickens, g** | | | **Growth rate** | | **NO oxidation, %^2^** |
| --- | --- | --- | --- | --- | --- | --- | --- | --- | --- |
|  |  |  |  | **1 day** | **14 days** | **28 days** | **2-week** | **4-week** |  |
| Broiler (Smena 8)^3,4^ | BRS | Meat | 64.8  ± 0.58 | 47.5  ± 0.7 | 311.9  ± 19.2 | 1157  ± 50 | 6.57 | 24.36 | 98.1  ± 2.5 |
| Broiler (Cobb 500)^3,4^ | BRC | Meat | 62.7  ± 0.67 | 48.5  ± 1.9 | 276.5  ± 17.5 | 1244.5  ± 38.4 | 5.70 | 25.66 | 97.8  ± 2.6 |
| Broiler (Ross 308)^3,4^ | BRR | Meat | 65.6  ± 0.7 | 43.4  ± 0.5 | 341.7  ± 10.1 | 1146.4  ± 30.5 | 7.87 | 26.41 | 97.4  ± 2.5 |
| Malay Game^3,4^ | MG | Game | 54.5  ± 0.45 | 37.9  ± 0.75 | 98.8  ± 4.4 | 214.8  ± 9.1 | 2.61 | 5.67 | 97.3  ± 2.6 |
| Uzbek Game (Kulangi)^3,4^ | UG | Game | 55.7  ± 0.55 | 41.3  ± 0.93 | 92.4  ± 3.8 | 223.7  ± 8.3 | 2.24 | 5.42 | 96.9  ± 2.6 |
| White Cornish | WC | Meat | 65.8  ± 0.6 | 49.3  ± 0.7 | 291.7  ± 9.4 | 1287.5  ± 49.1 | 5.92 | 26.12 | 96.9  ± 3.1 |
| Brahma Buff | BB | Dual purpose (egg-meat) | 60.4 | 38.2 | 107.5 | 241.7 | 2.81 | 6.33 | 74.1  ± 3.8 |
| Blue Meat-Egg Type (Andalusian) | BMET | Dual purpose (meat-egg) | 49.9  ± 0.6 | 40.1  ± 1 | 80.7  ± 3.2 | 231.8  ± 5.5 | 2.01 | 5.78 | 61.8  ± 2.9 |
| Plymouth Rock White | PRW | Dual purpose (meat-egg) | 64.3  ± 0.78 | 44.9  ± 0.68 | 265.2  ± 9.7 | 1058.4  ± 32.6 | 5.91 | 23.57 | 2.6  ± 1.5 |
| Layer (Hisex White)^3,4^ | LR | Egg | 64.2  ± 0.78 | 42.4  ± 3.1 | 79.8  ± 3.9 | 222.4  ± 5.2 | 1.88 | 5.25 | 2.4  ± 1.3 |
| Orloff Mille Fleur^3,4^ | OMF | Dual purpose (meat-egg) | 51.7  ± 0.5 | 35.5  ± 0.9 | 93.1  ± 5.8 | 167.8  ± 8.7 | 2.62 | 4.73 | 2.1  ± 1.5 |
| Andalusian Blue^3,4^ | AB | Dual purpose (meat-egg) | 48.5  ± 0.61 | 37.2  ± 0.97 | 85.4  ± 3.1 | 170.6  ± 5.4 | 2.30 | 4.59 | 2.1  ± 1.6 |
| Yurlov Crower^3,4^ | YC | Dual purpose (meat-egg) | 60.4  ± 0.7 | 39.1  ± 0.81 | 101.3  ± 4.5 | 240  ± 8.9 | 2.59 | 6.14 | <2 |

^1^Based on PCM, phenotypic clustering model (Larkina et al., 2021).

^2^ The degree of NO oxidation to nitrate is determined by the concentration ratio as follows: nitrate/(NO donors + nitrate) × 100%.

^3^Data from Dolgorukova et al., 2020.

^4^Data from Titov et al., 2020.

**Table S3.** Spearman correlation coefficient values in pairwise comparison of E7 NO oxidation rate (NO) and traits of early development and growth, including egg weight (EW) and body weight of chickens at 1-, 14- and 28-day-old (BW1, BW14 and BW28) in the 13 breeds studied.

| **Correlation/*p*-value^1^** | **EW** | **BW1** | **BW14** | **BW28** | **NO** |
| --- | --- | --- | --- | --- | --- |
| EW | 1 | 0.000400* | 0.003087* | 0.000876* | 0.096906 |
| BW1 | 0.833563* | 1 | 0.034031* | 0.000000* | 0.032936* |
| BW14 | 0.751032* | 0.598901* | 1 | 0.001423* | 0.022272* |
| BW28 | 0.806053* | 0.884615* | 0.807692* | 1 | 0.027221* |
| NO | 0.480001 | 0.592289* | 0.625347* | 0.608818* | 1 |

Spearman correlation coefficient values are given below the diagonal, and the corresponding *p*-values are above the diagonal. *Significant pairwise correlation values at *p* < 0.05.

**Table S4.** Spearman correlation coefficient values in pairwise comparison of DGE in the breast muscles and traits of early development and growth, including egg weight (EW) and body weight of chickens at 1-, 14- and 28-day-old (BW1, BW14 and BW28) in the eight breeds studied.

| **Correlation/ *p*-value** | **MSTN** | **GHR** | **MEF2C** | **MYOD1** | **MYOG** | **MYH1** | **MYF5** | **EW** | **BW1** | **BW14** | **BW28** |
| --- | --- | --- | --- | --- | --- | --- | --- | --- | --- | --- | --- |
| **MSTN** | 1 | 0.002232* | 0.002232* | 0.752034 | 0.427877 | 0.132292 | 0.881994 | 0.434639 | 0.976786 | 0.196627 | 0.243056 |
| **GHR** | 0.928571 | 1 | 0.002232* | 0.752034 | 0.840129 | 0.132292 | 0.664583 | 0.399264 | 0.934871 | 0.299206 | 0.299206 |
| **MEF2C** | 0.928571 | 0.928571 | 1 | 0.881994 | 0.500794 | 0.196627 | 0.976786 | 0.587845 | 0.881994 | 0.536409 | 0.582143 |
| **MYOD1** | -0.142857 | -0.142857 | -0.071429 | 1 | 0.069395 | 0.132292 | 0.299206 | 0.712762 | 0.840129 | 0.752034 | 0.752034 |
| **MYOG** | 0.333333 | 0.095238 | 0.285714 | 0.690476 | 1 | 0.536409 | 0.703323 | 0.691465 | 0.934871 | 0.389385 | 0.881994 |
| **MYH1** | 0.595238 | 0.595238 | 0.523810 | -0.595238 | -0.261905 | 1 | 0.934871 | 0.843546 | 0.793006 | 0.976786 | 0.619097 |
| **MYF5** | 0.071429 | -0.190476 | -0.023810 | -0.428571 | 0.166667 | -0.047619 | 1 | 0.452799 | 0.752034 | 0.934871 | 0.934871 |
| **EW** | 0.323359 | 0.347312 | 0.227549 | 0.155691 | 0.167668 | 0.083834 | -0.311383 | 1 | 0.001111 | 0.050063 | 0.007471 |
| **BW1** | 0.023810 | 0.047619 | -0.071429 | 0.095248 | 0.047619 | -0.119048 | -0.142857 | 0.922172 | 1 | 0.151141 | 0.036756 |
| **BW14** | 0.523810 | 0.428571 | 0.261905 | 0.142857 | 0.357143 | 0.023810 | -0.047619 | 0.706599 | 0.571429 | 1 | 0.004564 |
| **BW28** | 0.476191 | 0.428571 | 0.238095 | -0.142857 | 0.071429 | 0.214286 | -0.047619 | 0.850315 | 0.761905 | 0.904762 | 1 |

Spearman correlation coefficient values are given below the diagonal, and the corresponding *p*-values are above the diagonal. *Significant pairwise correlation values (at *p* < 0.05) as also highlighted with brown.

**Table S5.** Spearman correlation coefficient values in pairwise comparison of DGE in the thigh muscles and traits of early development and growth, including egg weight (EW) and body weight of chickens at 1-, 14- and 28-day-old (BW1, BW14 and BW28) in the eight breeds studied.

| **Correlation/ *p*-value** | **MSTN** | **GHR** | **MEF2C** | **MYOD1** | **MYOG** | **MYH1** | **MYF5** | **EW** | **BW1** | **BW14** | **BW28** |
| --- | --- | --- | --- | --- | --- | --- | --- | --- | --- | --- | --- |
| **MSTN** | 1 | 0.096181 | 0.299206 | 0.151141 | 0.955106 | 0.359871 | 0.243056 | 0.509054 | 0.881994 | 0.267460 | 0.170982 |
| **GHR** | 0.642857 | 1 | 0.703323 | 0.536409 | 0.887991 | 0.664583 | 0.021776 | 0.691465 | 0.752034 | 0.703323 | 0.582143 |
| **MEF2C** | 0.428571 | 0.166667 | 1 | 0.069395 | 0.332517 | 0.057589 | 0.216171 | 0.799460 | 0.326835 | 0.881994 | 0.703323 |
| **MYOD1** | 0.571429 | 0.261905 | 0.690476 | 1 | 0.026520 | 0.461806 | 0.243056 | 0.755833 | 0.840129 | 0.299206 | 0.619097 |
| **MYOG** | -0.023953 | -0.059881 | 0.395217 | 0.766481 | 1 | 0.777583 | 0.434639 | 0.743517 | 0.547943 | 0.490016 | 0.821452 |
| **MYH1** | 0.380952 | -0.190476 | 0.714286 | 0.309524 | -0.119763 | 1 | 0.976786 | 0.799460 | 0.752034 | 0.536409 | 0.752034 |
| **MYF5** | 0.476190 | 0.809524 | 0.500000 | 0.476191 | 0.323359 | -0.023810 | 1 | 0.168118 | 0.132292 | 0.840129 | 0.664583 |
| **EW** | 0.275454 | -0.167668 | -0.107786 | 0.131739 | -0.138554 | 0.107786 | -0.538932 | 1 | 0.001111 | 0.050063 | 0.007471 |
| **BW1** | 0.071429 | -0.142857 | -0.404762 | -0.095238 | -0.251502 | -0.142857 | -0.595238 | 0.9221722 | 1 | 0.151141 | 0.036756 |
| **BW14** | 0.452381 | 0.166667 | -0.071429 | 0.428571 | 0.287430 | -0.261905 | -0.095238 | 0.706600 | 0.571429 | 1 | 0.004563 |
| **BW28** | 0.547619 | 0.238095 | -0.166667 | 0.214286 | -0.095810 | -0.142857 | -0.190476 | 0.850315 | 0.761905 | 0.904762 | 1 |

Spearman correlation coefficient values are given below the diagonal, and the corresponding *p*-values are above the diagonal. *Significant pairwise correlation values (at *p* < 0.05) as also highlighted with brown.

**Table S6.** Spearman correlation coefficient values in pairwise comparison of the eight breeds based on data for 21 traits (DGE for myogenesis and NO metabolism in embryos, and early growth rates of chicks).

| **Correlation/ *p*-value** | **BR** | **UG** | **WC** | **BB** | **PRW** | **LR** | **OMF** | **YC** |
| --- | --- | --- | --- | --- | --- | --- | --- | --- |
| **BR** | 1 | 0.000313 | 0.006993 | 0.411923 | 0.001158 | 0.018071 | 0.000000 | 0.686204 |
| **UG** | 0.723377 | 1 | 0.034573 | 0.690377 | 0.054670 | 0.139383 | 0.001968 | 0.180101 |
| **WC** | 0.577922 | 0.466234 | 1 | 0.581174 | 0.013402 | 0.000014 | 0.001092 | 0.876678 |
| **BB** | 0.188312 | -0.092208 | 0.127273 | 1 | 0.438612 | 0.133044 | 0.753971 | 0.000124 |
| **PRW** | 0.671429 | 0.427273 | 0.536364 | 0.177922 | 1 | 0.018402 | 0.000060 | 0.204353 |
| **LR** | 0.515584 | 0.333766 | 0.802597 | 0.338961 | 0.514286 | 1 | 0.000094 | 0.200163 |
| **OMF** | 0.832468 | 0.646753 | 0.674026 | 0.072727 | 0.772727 | 0.761039 | 1 | 0.823572 |
| **YC** | -0.093506 | -0.303896 | -0.036364 | 0.753247 | 0.288312 | 0.290909 | 0.051948 | 1 |

Spearman correlation coefficient values are given below the diagonal, and the corresponding *p*-values are above the diagonal. *Significant pairwise correlation values (at *p* < 0.05) as also highlighted with pink.

**Table S7.** Spearman correlation coefficient values for pairwise comparison of DGE in the breast (b…) and thigh (t…) muscles and traits of early development and growth, including E7 oxidation of NO (NO), egg weight (EW) and body weight of chics at 1-, 14- and 28 days of age (BW1, BW14 and BW28) in the eight breeds studied.

| **Correlation/ *p*-value** | **bMSTN** | **bGHR** | **bMEF2C** | **bMYOD1** | **bMYOG** | **bMYH1** | **bMYF5** | **tMSTN** | **tGHR** | **tMEF2C** | **tMYOD1** | **tMYOG** | **tMYH1** | **tMYF5** | **NO** | **EW** | **BW1** | **BW14** | **BW28** | **GR2wk** | **GR4wk** |
| --- | --- | --- | --- | --- | --- | --- | --- | --- | --- | --- | --- | --- | --- | --- | --- | --- | --- | --- | --- | --- | --- |
| **bMSTN** | 1 | 0.002232 | 0.002232 | 0.752034 | 0.427877 | 0.132292 | 0.881994 | 0.010714 | 0.267460 | 0.045833 | 0.015377 | 0.399264 | 0.243056 | 0.243056 | 0.755833 | 0.434639 | 0.976786 | 0.196627 | 0.243056 | 0.619097 | 0.500794 |
| **bGHR** | 0.928571 | 1 | 0.002232 | 0.752034 | 0.840129 | 0.132292 | 0.664583 | 0.045833 | 0.299206 | 0.027927 | 0.083085 | 0.670344 | 0.196627 | 0.326835 | 0.821452 | 0.399264 | 0.934871 | 0.299206 | 0.299206 | 0.703323 | 0.840129 |
| **bMEF2C** | 0.928571 | 0.928571 | 1 | 0.881994 | 0.500794 | 0.196627 | 0.976786 | 0.069395 | 0.427877 | 0.004563 | 0.027927 | 0.416792 | 0.096181 | 0.243056 | 0.567772 | 0.587845 | 0.881994 | 0.536409 | 0.582143 | 0.934871 | 0.536409 |
| **bMYOD1** | -0.142857 | -0.142857 | -0.071429 | 1 | 0.069395 | 0.132292 | 0.299206 | 0.170982 | 0.045833 | 0.881994 | 0.582143 | 0.099101 | 0.976786 | 0.243056 | 0.712762 | 0.712762 | 0.840129 | 0.752034 | 0.752034 | 0.934871 | 0.196627 |
| **bMYOG** | 0.333333 | 0.095238 | 0.285714 | 0.690476 | 1 | 0.536409 | 0.703323 | 0.934871 | 0.427877 | 0.536409 | 0.045833 | 0.007471 | 0.840129 | 0.934871 | 0.547943 | 0.691465 | 0.934871 | 0.389385 | 0.881994 | 0.934871 | 0.021776 |
| **bMYH1** | 0.595238 | 0.595238 | 0.523810 | -0.595238 | -0.261905 | 1 | 0.934871 | 0.021776 | 0.326835 | 0.299206 | 0.619097 | 0.332517 | 0.083085 | 0.536409 | 0.332517 | 0.843546 | 0.793006 | 0.976786 | 0.619097 | 0.793006 | 0.793006 |
| **bMYF5** | 0.071429 | -0.190476 | -0.023810 | -0.428571 | 0.166667 | -0.047619 | 1 | 0.500794 | 0.151141 | 0.752034 | 0.536409 | 0.587845 | 0.427877 | 0.132292 | 0.887991 | 0.452799 | 0.752034 | 0.934871 | 0.934871 | 0.664583 | 0.881994 |
| **tMSTN** | 0.857143 | 0.738095 | 0.690476 | -0.547619 | 0.047619 | 0.809524 | 0.285714 | 1 | 0.096181 | 0.299206 | 0.151141 | 0.955106 | 0.359871 | 0.243056 | 0.865730 | 0.509054 | 0.881994 | 0.267460 | 0.170982 | 0.461806 | 0.752034 |
| **tGHR** | 0.452381 | 0.428571 | 0.333333 | -0.738095 | -0.333333 | 0.404762 | 0.571429 | 0.642857 | 1 | 0.703323 | 0.536409 | 0.887991 | 0.664583 | 0.021776 | 0.712762 | 0.691465 | 0.752034 | 0.703323 | 0.582143 | 0.752034 | 0.427877 |
| **tMEF2C** | 0.738095 | 0.785714 | 0.904762 | 0.071429 | 0.261905 | 0.428571 | -0.142857 | 0.428571 | 0.166667 | 1 | 0.069395 | 0.332517 | 0.057589 | 0.216171 | 0.191549 | 0.799460 | 0.326835 | 0.881994 | 0.703323 | 0.461806 | 0.461806 |
| **tMYOD1** | 0.833333 | 0.666667 | 0.785714 | 0.238095 | 0.738095 | 0.214286 | 0.261905 | 0.571429 | 0.261905 | 0.690476 | 1 | 0.026520 | 0.461806 | 0.243056 | 0.471261 | 0.755833 | 0.840129 | 0.299206 | 0.619097 | 0.934871 | 0.114980 |
| **tMYOG** | 0.347312 | 0.179644 | 0.335335 | 0.622766 | 0.850315 | -0.395217 | 0.227549 | -0.023953 | -0.059881 | 0.395217 | 0.766481 | 1 | 0.777583 | 0.434639 | 0.647409 | 0.743517 | 0.547943 | 0.490015 | 0.821452 | 0.509054 | 0.168118 |
| **tMYH1** | 0.476190 | 0.523810 | 0.642857 | -0.023810 | 0.095238 | 0.666667 | -0.333333 | 0.380952 | -0.190476 | 0.714286 | 0.309524 | -0.119763 | 1 | 0.976786 | 0.090890 | 0.799460 | 0.752034 | 0.536409 | 0.752034 | 0.976786 | 0.299206 |
| **tMYF5** | 0.476190 | 0.404762 | 0.476190 | -0.476190 | -0.047619 | 0.261905 | 0.595238 | 0.476190 | 0.809524 | 0.500000 | 0.476190 | 0.323359 | -0.023810 | 1 | 0.670344 | 0.168118 | 0.132292 | 0.840129 | 0.664583 | 0.196627 | 0.793006 |
| **NO** | -0.131739 | -0.095810 | -0.239525 | -0.155691 | -0.251502 | -0.395217 | 0.059881 | -0.071858 | 0.155691 | -0.514979 | -0.299407 | -0.192771 | -0.634742 | -0.179644 | 1 | 0.200729 | 0.099101 | 0.168118 | 0.090890 | 0.090890 | 0.068693 |
| **EW** | 0.323359 | 0.347312 | 0.227549 | 0.155691 | 0.167668 | 0.083834 | -0.311383 | 0.275454 | -0.167668 | -0.107786 | 0.131739 | -0.138554 | 0.107786 | -0.538932 | 0.506024 | 1 | 0.001111 | 0.050063 | 0.007471 | 0.002439 | 0.887991 |
| **BW1** | 0.023810 | 0.047619 | -0.071429 | 0.095238 | 0.047619 | -0.119048 | -0.142857 | 0.071429 | -0.142857 | -0.404762 | -0.095238 | -0.251502 | -0.142857 | -0.595238 | 0.622766 | 0.922172 | 1 | 0.151141 | 0.036756 | 0.015377 | 0.934871 |
| **BW14** | 0.523810 | 0.428571 | 0.261905 | 0.142857 | 0.357143 | 0.023810 | -0.047619 | 0.452381 | 0.166667 | -0.071429 | 0.428571 | 0.287430 | -0.261905 | -0.095238 | 0.538932 | 0.706599 | 0.571429 | 1 | 0.004563 | 0.057589 | 0.934871 |
| **BW28** | 0.476190 | 0.428571 | 0.238095 | -0.142857 | 0.071429 | 0.214286 | -0.047619 | 0.547619 | 0.238095 | -0.166667 | 0.214286 | -0.095810 | -0.142857 | -0.190476 | 0.634742 | 0.850315 | 0.761905 | 0.904762 | 1 | 0.007242 | 0.793006 |
| **GR2wk** | 0.214286 | 0.166667 | 0.047619 | -0.047619 | 0.047619 | 0.119048 | -0.190476 | 0.309524 | -0.142857 | -0.309524 | -0.047619 | -0.275454 | -0.023810 | -0.523810 | 0.634742 | 0.898220 | 0.833333 | 0.714286 | 0.880952 | 1 | 0.793006 |
| **GR4wk** | -0.285714 | -0.095238 | -0.261905 | -0.523810 | -0.809524 | -0.119048 | -0.071429 | -0.142857 | 0.333333 | -0.309524 | -0.619048 | -0.538932 | -0.428571 | 0.119048 | 0.670671 | -0.059881 | 0.047619 | -0.047619 | 0.119048 | 0.119048 | 1 |

Spearman correlation coefficient values are given below the diagonal, and the corresponding *p*-values are above the diagonal. *Significant pairwise correlation values (at *p* < 0.05) as also highlighted with pink.
