# Supplementary material for: Unraveling signatures of chicken genetic diversity and divergent selection in breed-specific patterns of early myogenesis, nitric oxide metabolism and post-hatch growth
Source: Front Genet. 2023 Jan 11;13:1092242. doi: 10.3389/fgene.2022.1092242 (PMC9874007; doi:10.3389/fgene.2022.1092242)
Supplement: Supplementary file 4 [file DataSheet2.docx]

Supplementary Material

Supplementary Information SI2: Complement to cluster analysis of the structure of differential gene expression

In the course of the further study based on the PCA method and using the ClustVis webtool, the primary values of the Type I FCs were also applied as variables (with their signs preserved; Tables 2 and 3). PCA plots (Figure SI2-1A,C), as well as heat maps (Figure SI2-1B,D), were obtained for the genes studied.

| **A** | **B** |
| --- | --- |

| **C** | **D** |
| --- | --- |

**Supplementary Figure SI2-1.** Analysis of the distribution of the seven genes according to the raw data (Type I) of their DGE in the breast **(A, B)** and thigh **(C, D)** muscles among the eight breeds as performed in the ClustVis program (Metsalu and Vilo, 2015). **(А, C)** PCA plots. X and Y axes show principal component 1 (PC1) and principal component 2 (PC2) that explain 51.1% and 31.7% **(A)**, and 37.8% and 30.5% **(C)** of the total variance, respectively. N = 7 data points (genes). **(B, D)** Heatmaps and clustering trees plotted using Euclidean distances (with the average option selected as linkage method).

Figure SI2-1 shows that the *GHR* and *MSTN* genes have consistent clustering patterns, and, therefore, DGE in all breeds and in both (breast and thigh) types of muscle tissue; *MYH1* is close to them and then *MYOD1*. The remaining genes have peculiar DGE profiles in different breeds, but in general for the breast muscles (Figure SI2-1A), there is a similar pattern of distribution of the seven genes observed on the PCA plot as that on the vector plot (Figure 1A), including the combined direction of DGE of the *MYOG* and *MYOD1* genes. On the graphs for the thigh muscles, one can also see a certain similarity between point and vector patterns of DGE of the examined genes.

Further, so that the clustering algorithm does not depend on the value of the units of raw variables, the data were scaled (standardized) using the scale function in R (Wickham et al., 2020). Using this transformed dataset (Type IIa) and hierarchical clustering using UPGMA performed with the R package pvclust (Suzuki and Shimodaira, 2006), the corresponding trees were constructed for the genes differentially expressed in the breast (Figure SI2-2A,B) and thigh (Figure SI2-2C,D) muscles.

| **A** | **B** |
| --- | --- |

| **C** | **D** |
| --- | --- |

**Supplementary Figure SI2-2.** Hierarchical clustering of the seven genes differentially expressed in the breast **(A, B)** and thigh **(C, D)** muscles and assessed for FC level using the R package pvclust (Suzuki and Shimodaira, 2006) and based on the Type IIa dataset. Clustering was carried out using the UPGMA method coupled with bootstrapping (10,000 iterations). **(A, C)** Clustering results are presented in the tree form. Values on the dendrogram are AU p-values (red, left), BP values (green, right), and cluster labels 1–5 (grey, bottom). Clusters with AU ≥ 95% are indicated by the rectangles and are considered to be strongly supported by data. **(B, D)** Triangle plots.

Using this approach (Figure SI2-2), it was also found that the *MSTN*, *GHR*, and *MYOD1* genes formed a tightly clustered group of genes.

The agglomeration coefficient for the breast and thigh muscle genes in Figure SI2-2 was 0.8530324 and 0.708165, respectively, with the respective clustering method selected.

Testing the optimal number of clusters (Figure SI2-3) indicated the presence of five clusters.

| **A** | **B** |
| --- | --- |

**Supplementary Figure SI2-3.** Choosing the optimal number of clusters *k* using the elbow method (Zhao et al., 2008) for breast **(A)** and thigh **(B)** muscle genes, which was close to *k* = 5, although no clear inflection was observed, meaning that the number of clusters could vary between 5 and 6.

Thus, the *MSTN*, *MYOD1*, and *GHR* genes formed a separate cluster. The remaining genes formed a chain of genes that do not have branching.

As a result of the obtained correlation values of the *MSTN*, *GHR* and *MYOD1* genes, *GHR* was selected in broiler chickens for the purpose of its detailed consideration. *GHR* has less variability as compared to *MSTN* and *MYOD1*.
