# Supplementary material for: Unraveling signatures of chicken genetic diversity and divergent selection in breed-specific patterns of early myogenesis, nitric oxide metabolism and post-hatch growth
Source: Front Genet. 2023 Jan 11;13:1092242. doi: 10.3389/fgene.2022.1092242 (PMC9874007; doi:10.3389/fgene.2022.1092242)
Supplement: Supplementary file 5 [file DataSheet1.docx]

Supplementary Material

Supplementary Information SI1: Details for the Type II and III transformed datasets

# Type IIa and IIb datasets

In order not to depend on the unit dimension of the raw FC variables and to generate a more suitable Type IIa dataset, we scaled (standardized) the raw data using the scale function in R (Wickham et al., 2020) that resulted in Type IIa dataset. Negative FC values were corrected by mathematical transformation 1/log_2_(–FC). Alternatively, the Adjust function and the Log 2 and Quantile normalize options in the Phantasus web application (Zenkova et al., 2018) were used for visual and interactive analysis of DGE, and the Type IIb dataset was then obtained.

# Type IIIa and IIIb datasets

To enhance the resolution of the analysis using the DGE data, the following transformation was undertaken based on the calculation of multiplicity factors of pairwise FC values between breeds. For this, the total number of raw FC values for each breed and for each gene, i.e., 56 of these values (eight breeds by seven genes), were used to calculate pairwise multiple ratios of FC scores for any two breeds and for each particular gene. Thus, the resulting pairwise multiple ratios of FC, or their multiplicity factors, showed how many times the expression of an individual gene in one breed exceeds that in another breed. At the same time, seven intermediate tables (matrices) were produced for such pairwise multiple ratios for the eight studied breeds, i.e., according to the number of analyzed genes, of which we had seven.

At the next stage, a certain generalizing indicator of pairwise ratios for all seven genes was obtained using two different approaches: (a) calculating pairwise Euclidean metrics for seven genes, taking pairwise FC ratios for single genes; and (b) deriving average pairwise FC ratios across seven genes. As a result, two matrices of pairwise FC ratios were obtained: one based on Euclidean metrics (Type IIIa), and the other based on averaged pairwise FC ratios (Type IIIb).
